# HTGTS‐TCR‐Seq for Profiling of Mouse and Human T‐Cell Receptor α and β Gene Rearrangements and Diversity

**DOI:** 10.1002/advs.202509497

**Published:** 2025-10-27

**Authors:** Rui Luo, Yawei Song, Meichen Wang, Longhao Zou, Fangtai Jiao, Tiange Yang, Guangchuan Wang, Zhuoyi Liang, Wei Wu, Hai‐Qiang Dai

**Affiliations:** ^1^ State Key Laboratory of Epigenetic Regulation and Intervention CAS Key Laboratory of Multi‐Cell Systems Shanghai Institute of Biochemistry and Cell Biology Center for Excellence in Molecular Cell Science Chinese Academy of Sciences University of Chinese Academy of Sciences Shanghai 200031 China; ^2^ First Affiliated Hospital of Chongqing Medical University Chongqing Medical University Chongqing 400016 China; ^3^ Bioscience and Biomedical Engineering Thrust Brain and Intelligence Research Institute The Hong Kong University of Science and Technology (Guangzhou) Guangzhou 511453 China

**Keywords:** αβ T cells, antigen receptor diversification, TCR repertoire, HTGTS‐TCR‐seq, V(D)J recombination

## Abstract

Developing αβ T lymphocytes generate T‐cell receptor (TCR) diversity through V(D)J recombination, which assembles *Tcra* and *Tcrb* genes from germline variable (V), diversity (D), and joining (J) segments. Approaches to characterize TCR rearrangements and diversity are critical for studying T‐cell development and immune function. Several existing methods, such as multiplex PCR and 5′RACE, have advanced the field; however, each carries inherent technical limitations. Here, high‐throughput Genome‐wide translocation sequencing‐based TCR sequencing (HTGTS‐TCR‐seq), a complementary and cost‐effective strategy for quantitative profiling of *Tcra* and *Tcrb* gene rearrangements, is presented. HTGTS‐TCR‐seq employs a limited set of 3–5 J or V region primers to enrich for V(D)J recombination products, allowing detection of both productive and nonproductive rearrangements. Application to wild‐type murine thymocytes at defined developmental stages, as well as young and aged T cells, reveals stage‐specific V and J usage and age‐associated repertoire alterations. Analysis of *Wapl*‐knockout preselection double‐positive thymocytes uncovers a cell division‐independent role for the cohesin‐unloading factor WAPL in *Tcra* rearrangement. Moreover, analysis of human peripheral T cells demonstrates conserved complementarity‐determining region 3 (CDR3) features and subset‐specific Vβ usage across species. Collectively, HTGTS‐TCR‐seq provides an efficient and accessible approach for quantifying TCR rearrangements and diversity across development, aging, and immune‐related conditions.

## Graphical abstract

HTGTS‐TCR‐seq is an efficient and cost‐effective method for profiling TCRα and TCRβ rearrangements using only a few primers. It captures both productive and nonproductive recombination events, minimizes primer bias, and reveals stage‐specific and age‐related repertoire changes in mice, as well as conserved features in human peripheral T cells.

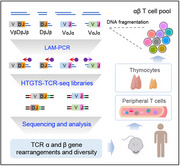

## 1 Introduction

The T‐cell receptor (TCR) is a fundamental component of the adaptive immune system, enabling T cells to recognize diverse antigens presented by major histocompatibility complex (MHC) molecules. In both humans and mice, TCR diversity is generated through V(D)J recombination, a process that assembles variable (V), diversity (D), and joining (J) gene segments into functional TCR α and β chains. During recombination, random nucleotide insertions and deletions at the V(D)J junctions give rise to highly variable complementarity‐determining region 3 (CDR3) sequences, the most diverse and antigen‐contacting part of the TCR.^[^ ^1^ ^]^ In αβ T cells, the combinatorial V(D)J joining and the intrinsic imprecision of coding join formation together contribute to the vast diversity of *Tcra* and *Tcrb* repertoires.

In mice, the *Tcrb* locus spans ≈685 kilobases (kb) and contains multiple Vβ and Jβ segments, as well as two Dβ segments. In contrast, the *Tcra* locus extends over ≈1.8 megabases (Mb) and comprises a large number of Vα and Jα segments.^[^ ^2^ ^]^ These loci undergo sequential and developmentally regulated rearrangements during thymocyte maturation. In the thymus, *Tcrb* recombination initiates at the CD4^−^CD8^−^ (double negative or DN) stage with Dβ‐to‐Jβ, followed by Vβ‐to‐DβJβ joining.^[^ ^3^ ^]^ This process is tightly regulated both in cis and in trans between the two alleles. Vβ‐to‐DβJβ rearrangement occurs asynchronously between alleles, and the expression of a functional TCRβ chain from one allele provides feedback inhibition that suppresses further Vβ recombination on the other allele, thereby ensuring allelic exclusion.^[^ ^4^ ^]^ Productive TCRβ expression promotes β‐selection, leading to proliferation and differentiation of DN thymocytes into CD4⁺CD8⁺ double‐positive (DP) cells. At the DP stage, *Tcra* recombination is initiated through Vα‐to‐Jα joining. In contrast to *Tcrb*, *Tcra* rearrangement is not subject to allelic exclusion and typically occurs on both alleles. The recombination process involves multiple rounds of primary and secondary rearrangements. Initial recombination events preferentially utilize Vα segments located near the 3′ end of the Vα array and Jα segments close to the 5′ end of the Jα array near the recombination center (RC). Subsequent rounds of rearrangement progressively engage more distal Vα and Jα segments in a stepwise fashion. This ordered progression is regulated by cis‐acting regulatory elements and is influenced by prior *Tcrd* rearrangement events that occur during the DN stage.^[^ ^5^, ^6^ ^]^ This iterative process continues until a functional TCRαβ complex is formed and the cell undergoes positive selection, or it is eliminated by apoptosis.^[^ ^7^ ^]^ Positively selected thymocytes further differentiate into CD4⁺ or CD8⁺ single‐positive (SP) T cells, which exit the thymus as naïve, mature αβ T cells, or eliminated by apoptosis during negative selection.^[^ ^8^ ^]^

While V(D)J junctional diversity is essential for immune defense, it presents substantial challenges for analyzing *Tcra* and *Tcrb* gene rearrangements.^[^ ^9^, ^10^, ^11^, ^12^ ^]^ Traditional approaches, such as multiplex PCR, are used at both DNA and mRNA levels. These methods require large sets of primers targeting V and J segments, but are inherently limited by primer preference bias.^[^ ^13^, ^14^, ^15^ ^]^ The 5′Rapid Amplification of cDNA Ends (5′RACE) technique offers a straightforward approach to detect TCR transcripts using a single primer pair targeting the common adapter and constant region.^[^ ^16^ ^]^ While this method avoids primer bias, obtaining and preserving mRNA samples are relatively challenging. Additionally, 5′RACE is incapable of detecting rearrangement at both alleles involving allelic exclusion, thereby limiting its application in comprehensively describing TCR rearrangement events. The advent of single‐cell RNA sequencing and spatial transcriptomics has enabled single‐cell paired TCR sequencing, providing enhanced functional insights into TCR diversity. However, these approaches are constrained by high cost and limited scalability.^[^ ^17^, ^18^, ^19^, ^20^ ^]^ Target enrichment strategies have also been employed to capture specific TCR sequences from bulk samples.^[^ ^21^ ^]^ In the B cell field, techniques such as HTGTS‐rep‐seq (an adaptation of LAM‐HTGTS) and VDJ‐seq have been used to profile immunoglobulin rearrangements with minimal bias using J segment primers and DNA‐based capture.^[^ ^22^, ^23^, ^24^ ^]^ Despite these advances, no comparable DNA‐based method has been broadly adopted for unbiased and quantitative analysis of TCRα and TCRβ repertoires.

In this study, we describe HTGTS‐TCR‐seq, a targeted sequencing approach that uses 3–5 J or V primers to enable unbiased capture of recombined *Tcra* and *Tcrb* segments. We applied this method to murine T cell populations at defined developmental and aging stages, including DN3, preselection DP, total thymocytes, peripheral CD4⁺ and CD8⁺ T cells, as well as *Cd4*‐Cre‐mediated *Wapl*‐knockout DP thymocytes. We further extended the approach to human peripheral CD4⁺ and CD8⁺ T cells isolated from peripheral blood mononuclear cells (PBMCs). Together, HTGTS‐TCR‐seq provides an efficient and accessible approach for profiling TCRα and TCRβ repertoires through T cell development, aging, and immune contexts.

## 2 Results

### 2.1 Overview of HTGTS‐TCR‐Seq for Profiling of TCR α and β Gene Rearrangements

For HTGTS‐TCR‐seq libraries, we employed bait sequences targeting coding ends of J or V segments of *Tcra* and/or *Tcrb* loci to profile mouse TCR α‐ and β‐chain diversity unbiasedly in thymocytes and periphery T cells. Both productive and nonproductive rearrangements were also included, providing a direct overview of functional and nonfunctional rearrangements within each T cell population (Figure S1A, Supporting Information). Genomic DNA from each sample was sonicated to generate fragments averaging 0.7 kb in length, sufficient to encompass V(D)J rearrangements as well as unrearranged J segments. Biotinylated primers designed to anneal downstream of the coding ends enabled linear amplification of fragments containing the targeted bait regions (Figure S1B, Supporting Information). Streptavidin bead purification, ligation of adapters, nested PCR for barcode incorporation, and library construction were performed following established protocols (Figure S1C–E, Supporting Information).^[^ ^24^ ^]^ Libraries were sequenced using paired‐end 150‐bp reads on an Illumina NovaSeq platform. TCR rearrangements were analyzed using an IgBLAST‐based pipeline, which provided detailed annotation of productive and nonproductive junctions, including CDR3 sequences based on conserved cysteine and phenylalanine residues in TCR V and J segments (Figure S1A, Supporting Information).^[^ ^25^ ^]^

### 2.2 HTGTS‐TCR‐Seq Reveals *Tcrb* Gene Rearrangements in Developing Mouse Thymocytes

**Figure 1:**
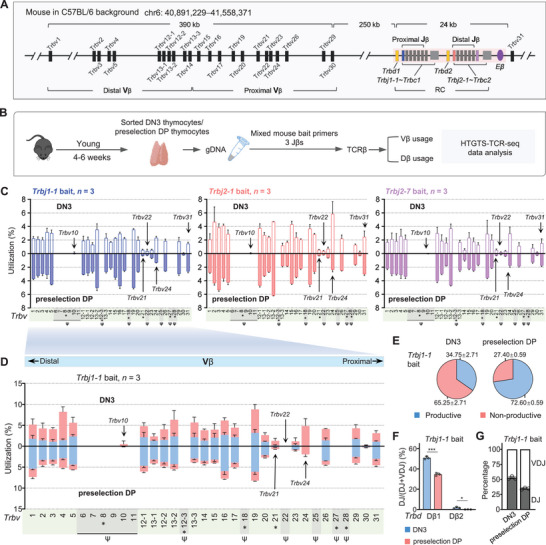
HTGTS‐TCR‐seq analysis of *Tcrb* gene rearrangement in DN3 and preselection DP thymocytes from young C57BL/6 mice. A) Schematic representation of the murine *Tcrb* locus, illustrating the locations of Vβ, Dβ, Jβ, and Cβ segments. The positions of three Jβ bait primers (*Trbj1‐1*, *Trbj2‐1*, and *Trbj2‐7*) used for HTGTS‐TCR‐seq in this study are highlighted with different colors. Data presented are derived from IMGT. B) Outline of the HTGTS‐TCR‐seq workflow for profiling the TCRβ repertoire in sorted DN3 and preselection DP thymocytes of young C57BL/6 mice. Genomic DNA (gDNA) was extracted and subsequently employed with the indicated bait primers for library preparation. C) Comparison analysis of the Vβ segment usage within *Tcrb* gene rearrangements in DN3 (up) and preselection DP (down) using three different bait primers: *Trbj1‐1* (left), *Trbj2‐1* (middle), and *Trbj2‐7* (right). Pseudogenes are denoted by ψ with gray background shading, and gene segments with nonfunctional RSSs are marked with an asterisk. D) Vβ repertoire profiles with productive and nonproductive information from VβDβJβ rearrangements in DN3 (up) and preselection DP (down) thymocytes, using Jβ bait primer *Trbj1‐1*. E) Pie charts showing the average overall percentage of productive versus nonproductive VβDβJβ rearrangements derived from libraries shown in panels (D) for DN3 (left) and preselection DP (right) thymocytes. F) Comparison analysis of Dβ segment usage within DβJβ rearrangements in DN3 (blue) and preselection DP (red), as revealed with bait primer *Trbj1‐1*. *P* values were calculated via Student′s *t*‐test; ^*^ *p* ≤ 0.05, ^**^ *p* ≤ 0.01, and ^***^ *p* ≤ 0.001. G) Comparison of DβJβ: VβDβJβ ratios in sorted DN3 (left) and preselection DP (right), determined using bait primer *Trbj1‐1*. Shown are the average utilization frequencies ± s.d. of all Vβ segments from 3 independent C57BL/6 mice. *n*, number of mice. See Tables S1 and S2 and Figure S2 (Supporting Information) for more information.

The *Tcrb* locus in C57BL/6 mice comprises 35 *Vβ* **segments, of which 23** Vβs are annotated as functional. It also contains two DβJβ clusters: each consists of a single Dβ segment (*Trbd1* or *Trbd2*) and seven Jβ segments (*Trbj1‐1* to *1‐7* or *Trbj2‐1* to *2‐7*) (**Figure** **1**A). Among these, *Trbj1‐7* and *Trbj2‐6* are nonfunctional. To evaluate the ability of HTGTS‐TCR‐seq in detecting stage‐specific *Tcrb* gene rearrangements during thymocyte development, we purified primary CD4^−^CD8^−^CD44^−^CD25^+^ DN3 thymocytes and CD4^+^CD8^+^CD69^−^TCRβ^lo^ preselection DP thymocytes from the thymus of wild‐type (WT) young C57BL/6 mice (Figure 1B). These two populations represent sequential stages of T cell development, before and after successful β‐selection, and are critical for assessing the dynamics of *Tcrb* recombination. Genomic DNA from each population was subjected to HTGTS‐TCR‐seq using three different coding‐end *Jβ* bait primers (*Trbj1‐1*, *Trbj2‐1*, and *Trbj2‐7*) to comprehensively capture *VβDβJβ* and *DβJβ* rearrangements (Figure S2A,B, Supporting Information).

HTGTS‐TCR‐seq revealed highly reproducible Vβ usage patterns across three biological replicates and among different Jβ bait primers. Libraries from both DN3 and preselection DP thymocytes exhibited broad yet nonrandom distributions of *Vβ* segment usage across the *Trbv* locus, with certain segments more frequently used (Figure 1C). HTGTS‐TCR‐seq detected all 23 functional *Vβ* segments *within VβDβJβ* exons, as well as two pseudogene *Vβ* segments (*Trbv10* and *Trbv22*) in both DN3 and preselection DP thymocytes. Analysis of productive rearrangements revealed that 22 of the 23 functional Vβ segments were used at relatively comparable frequencies, while *Trbv30* was notably underutilized (Figure 1D; Figure S2C,D, Supporting Information). *Trbv21* and *Trbv24* were predominantly observed in nonproductive rearrangements, indicating limited contribution to the functional TCRβ repertoire. Although pseudogene *Trbv22* was detected in recombination events, it was absent from the productive rearrangement pool (Figure 1C, D). Interestingly, pseudogene *Trbv10* was still detectable in low‐frequency unproductive rearrangements in DN3 thymocytes.

*Vβ‐to‐DβJβ* rearrangements occur at the DN3 stage. Due to the imprecision of coding join formation, only one‐third of rearrangements are expected to be in‐frame. Using *Trbj1‐1* as the bait primer, HTGTS‐TCR‐seq revealed that, on average, 34.8% of Vβ‐to‐DβJβ rearrangements in DN3 thymocytes were in‐frame, closely matching the theoretical estimate of ≈33% (Figure 1E).^[^ ^26^ ^]^ In contrast, 72.6% of rearrangements in preselection DP thymocytes were in‐frame, consistent with the expected enrichment (≈71.4%) of productive TCRβ rearrangements following β‐selection.^[^ ^14^ ^]^ Similar results were obtained with the other two Jβ bait primers (Figure S2E,F, Supporting Information). These ratios likely reflect both regulation of Vβ rearrangements between alleles and the requirement for TCRβ protein expression to drive thymocyte progression from the DN3 to DP stage.^[^ ^27^ ^]^ Analysis of DβJβ junctions revealed distinct recombination patterns between the two Dβ segments: *Trbd1* segment exhibited dual recombination potential with both Jβ1 and Jβ2 clusters, whereas *Trbd2* recombined exclusively with Jβ2 cluster (Figure 1F; Figure S2G,H, Supporting Information). Moreover, we observed a significantly higher frequency of DβJβ rearrangements in DN3 thymocytes compared to preselection DP thymocytes across all Jβ bait primers (Figure 1G; Figure S2I,J, Supporting Information), consistent with the model in which Dβ‐to‐Jβ rearrangements on both alleles precede VβDβJβ recombination at the DN3 stage.^[^ ^28^ ^]^ These results demonstrate that HTGTS‐TCR‐seq captures both productive and nonproductive *Tcrb* rearrangements, enabling detailed characterization of Vβ usage and recombination dynamics with high detection capability and developmental stage specificity during early T cell development.

### 2.3 HTGTS‐TCR‐Seq Detects *Tcra* Gene Rearrangements in Mouse Preselection DP Thymocytes

**Figure 2:**
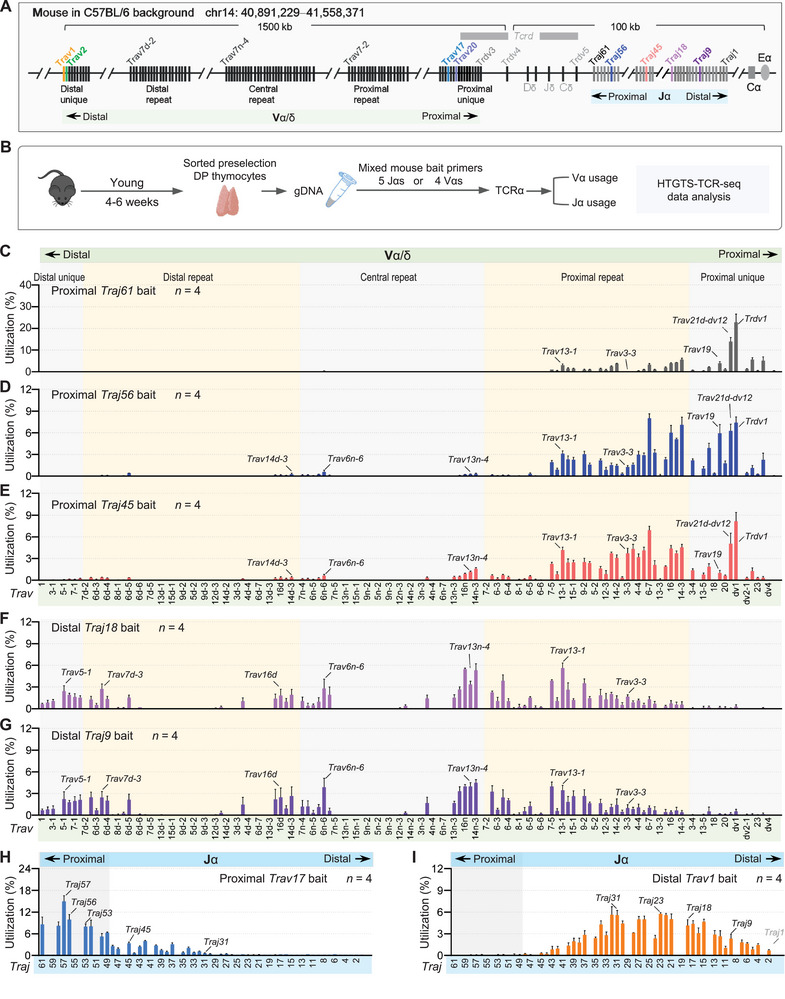
HTGTS‐TCR‐seq analysis of *Tcra* gene rearrangement in sorted preselection DP thymocytes from young C57BL/6 mice. A) Schematic representation of the murine *Tcra‐Tcrd* locus, illustrating the locations of Vα and Jα gene segments. The positions of five Jα bait primers (*Traj61*, *Traj56*, *Traj45*, *Traj18*, and *Traj9*) and four Vα bait primers (*Trav20*, *Trav17*, *Trav2*, and *Trav1*) used for HTGTS‐TCR‐seq in this study are highlighted with different colors. Data presented are derived from IMGT. B) Overview of the HTGTS‐TCR‐seq workflow used to profile the TCRα repertoire in sorted preselection DP thymocytes from young C57BL/6 mice. gDNA was extracted from sorted preselection DP thymocytes and subsequently employed with indicated bait primers for library preparation. C–G) Vα segment usage in VαJα rearrangements in sorted preselection DP thymocytes using proximal Jα bait primers *Traj61* (C), *Traj56* (D), and *Traj45* (E), and distal Jα bait primers *Traj18* (F) and *Traj9* (G), respectively. Data represent the average utilization frequencies ± s.d. of all Vα segments from 4 independent C57BL/6 mice. H,I) Jα segment usage in VαJα rearrangements in sorted preselection DP thymocytes using proximal Vα bait primer *Trav17* (H) and distal Vα bait primer *Trav1* (I). Shown are the average utilization frequencies ± s.d. of all Jα segments from 4 independent C57BL/6 mice. For comparison, several Vα and Jα segments are indicated. *n*, number of mice. See Table S2 and Figure S2 (Supporting Information) for more information.

The *Tcra* locus in C57BL/6 mice contains 138 Vα and 60 Jα segments, of which 117 Vα and 43 Jα segments are functionally rearrangeable (**Figure** **2**A). This region also includes the *Tcrd* locus, which undergoes rearrangement at the DN stage and contributes to the diversification of the TCRα repertoire.^[^ ^5^, ^6^ ^]^ Given the large number of Jα segments and the complex, repetitive architecture of the *Tcra* locus, where many Vα family members are highly similar or even identical, it is not feasible to capture the full TCRα repertoire with a single Jα bait primer.

To first assess the performance of HTGTS‐TCR‐seq in detecting *Tcra* gene rearrangements, we analyzed sorted preselection DP thymocytes from WT young C57BL/6 mice (Figure 2B). These cells reflect the intrinsic rearrangement potential of the *Tcra* locus before positive or negative thymic selection. We employed five Jα‐targeting bait primers spanning distinct regions of the *Tcra* locus, including proximal (*Traj61*, *Traj56*, and *Traj45*) and distal (*Traj18* and *Traj9*) Jα segments, to examine Vα utilization patterns (Figure 2A; Figure S2K, Supporting Information). Although the first proximal Jα segment *Traj61* is annotated as a pseudogene, HTGTS‐TCR‐seq detected extensive rearrangements only to the very proximal Vα segments, including parts of the proximal repeat Vα array (Figure 2C). In contrast, the functional proximal Jα segments *Traj56* and *Traj45* not only recombined extensively with Vα segments in both proximal unique and proximal repeat regions, but also exhibited broader Vα rearrangements across the entire locus, albeit at much lower frequencies in the central repeat and distal repeat regions (Figure 2D, E). Notably, we observed multiple clusters of Vα segments within the central to distal repeat regions beginning to rearrange to *Traj56* or *Traj45*, suggesting spatial regulation of locus accessibility. With more distal Jα baits such as *Traj18* and *Traj9*, Vα usage progressively shifted toward the central to distal Vα locus (Figure 2F, G). Furthermore, using proximal Jα bait primers, we also detected high‐frequency recombination of Vδ segments, particularly *Trav21‐dv12* and *Trdv1*, whereas the inversional *Trdv5* was not observed (Figure 2C–E).

To further characterize Jα usage, we employed bait primers targeting four Vα segments, including proximal (*Trav20* and *Trav17*) and distal (*Trav2* and *Trav1*) Vα regions (Figure 2A; Figure S2K, Supporting Information). Although *Trav20* is annotated as a pseudogene, the Jα usage pattern detected using the *Trav20* bait closely resembled that observed with the functional *Trav17* bait. Both baits revealed predominant usage of proximal Jα segments, with *Traj57* being the most frequently utilized, followed by a gradual decline in usage toward more distal Jα segments (Figure 2H; Figure S2L, Supporting Information). Using distal *Trav2* and *Trav1* baits, we observed replacement of proximal Jα segments by middle and distal ones, resulting in a Gaussian‐like distribution of Jα usage centered around the mid‐cluster (Figure 2I; Figure S2M, Supporting Information). Collectively, these data demonstrate that HTGTS‐TCR‐seq provides an effective and reproducible approach for delineating *Tcra* gene rearrangements in developing thymocytes.

### 2.4 Validation of HTGTS‐TCR‐Seq

To benchmark HTGTS‐TCR‐seq against established TCR‐sequencing methods, we first compared it with 5′RACE using RNA and DNA from total thymocytes of young adult mice.^[^ ^14^ ^]^ Both approaches produced highly consistent Vβ usage frequencies across *Tcrb* locus (Figure S3A,B, Supporting Information). While HTGTS‐TCR‐seq largely recapitulated the 5′RACE profiles of *Tcrb* gene rearrangements, several Vβ segments (e.g., Vβ2, Vβ12‐1, Vβ19, Vβ23) displayed notable differences, likely reflecting transcript‐level variation (Figure S3A,B, Supporting Information). For the *Tcra* locus, HTGTS‐TCR‐seq similarly captured overall Jα usage patterns with representative proximal and distal *Trav* baits but, in contrast to 5′RACE, uniquely detected rearrangements to pseudogene segments such as Jα61, which were nearly undetectable by 5′RACE (Figure S3C, Supporting Information). Moreover, compared with 5′RACE assay, HTGTS‐TCR‐seq revealed distinct distributions at a subset of Vα recombination events at the DNA level (Figure S3D–G, Supporting Information). These features, consistent with the stepwise nature of *Tcra* gene recombination, demonstrate that transcript‐based profiles may not always faithfully represent recombination frequency, whereas HTGTS‐TCR‐seq could provide a more direct view of repertoire dynamics.

For a broader context, we also summarized comparisons of HTGTS‐TCR‐seq with multiplex PCR, 5′RACE, and single‐cell TCR‐seq (Table S4, Supporting Information).^[^ ^29^, ^30^, ^31^, ^32^, ^33^, ^34^, ^35^, ^36^, ^37^ ^]^ Although multiplex PCR is also a DNA‐based bulk approach, it has so far been limited to TCRβ repertoire analysis and requires >24 paired primers and multiple reactions to quantify individual Vβ‐to‐DβJβ rearrangements, with careful optimization to avoid primer‐associated amplification bias. In contrast, HTGTS‐TCR‐seq relies on linear amplification from a single or a few bait primers at the *Tcra* and *Tcrb* loci, reducing amplification bias and enabling unbiased capture of rearrangements irrespective of gene segment usage. HTGTS‐TCR‐seq combines the breadth and reproducibility of bulk approaches with the ability to interrogate DNA‐level recombination events, including pseudogenes, thereby offering complementary advantages for analyzing TCR rearrangements and repertoire diversity.

### 2.5 Analysis of Age‐Related Alterations in TCR Repertoire in the Thymus

**Figure 3:**
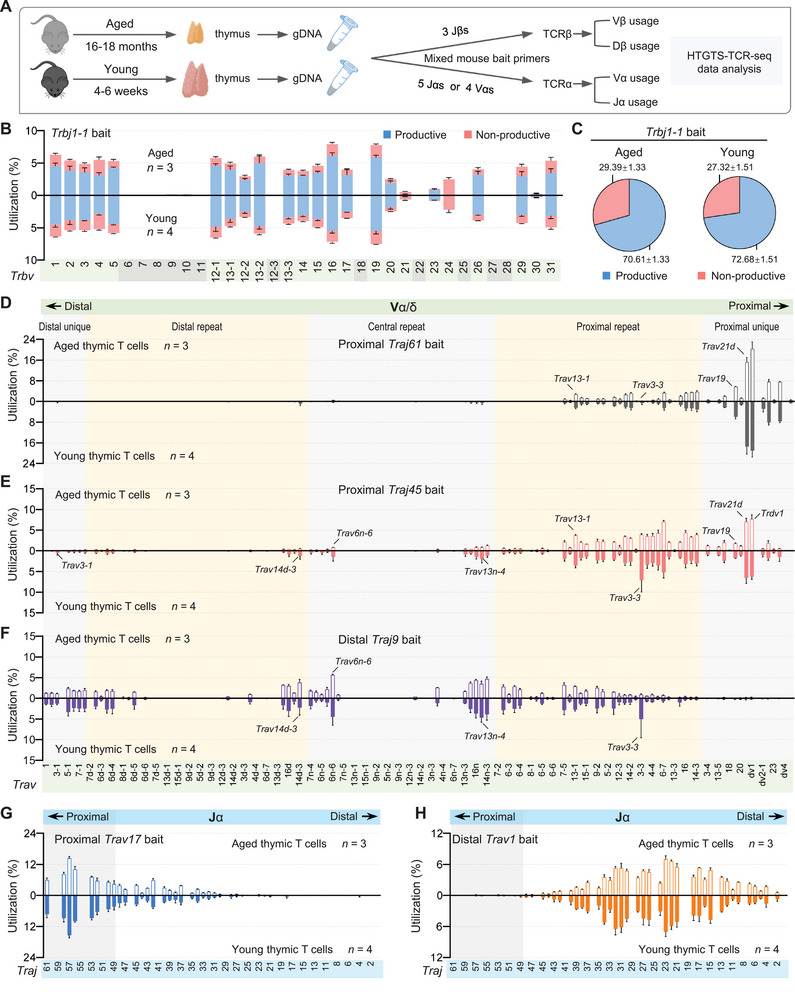
Comparison analysis of TCR repertoires in aged and young thymocytes of C57BL/6 mice. A) Overview of the HTGTS‐TCR‐seq experimental design for profiling the TCRβ and TCRα repertoires in total thymocytes from aged and young C57BL/6 mice. gDNA was extracted from total thymocytes and subsequently employed with indicated bait primers for library preparation. B) Vβ repertoire profiles with productive and nonproductive information from VβDβJβ rearrangements in aged (up) and young (down) thymocytes, using Jβ bait primer *Trbj1‐1*. Pseudogenes are denoted with gray background shading. C) Pie charts showing the average overall percentage of productive versus nonproductive VβDβJβ rearrangements derived from libraries shown in panel (B) for aged (left) and young (right) thymocytes. D–F) Vα segment usage in VαJα rearrangements from aged (up) and young (down) thymocytes using proximal Jα bait primers *Traj61* (D), *Traj45* (E), and distal Jα bait primers *Traj9* (F). For comparison, several Vα segments are highlighted. G,H) Jα segment usage in VαJα rearrangements in aged (up) and young (down) thymocytes using proximal Vα bait primer *Trav17* (G) and distal Vα bait primer *Trav1* (H). Data are presented as mean ± s.d.; *n*, number of mice. See Table S2 and Figure S4 (Supporting Information) for more information.

Thymic involution is a hallmark of vertebrate immunosenescence, progressing with age and leading to reduced output of naïve T cells and diminished peripheral TCR diversity and function.^[^ ^38^, ^39^ ^]^ To test whether HTGTS‐TCR‐seq can effectively assess age‐related changes in the thymic TCR repertoire during this process, we performed HTGTS‐TCR‐seq on thymocytes isolated from young (4–6 weeks) and aged (16–18 months) C57BL/6 mice (**Figure** **3**A). This analysis enabled a direct comparison of age‐dependent alterations in *Tcra* and *Tcrb* gene rearrangements and diversity.

TCRβ repertoire analysis revealed broadly conserved Vβ segment usage patterns between young and aged thymocytes across different Jβ bait primers, although subtle differences in segment preference were observed depending on the specific bait primer (Figure 3B; Figure S4A–C, Supporting Information). *Trbv24*, although annotated as functional, was predominantly involved in nonproductive rearrangements in both age groups. Notably, aged thymocytes showed a modest but consistent reduction in the proportion of productive VβDβJβ rearrangements across all baits (Figure 3C; Figure S4D,E, Supporting Information). In contrast, the relative frequencies of Dβ usage, as well as the ratio of VβDβJβ to DβJβ rearrangement, remained largely unchanged between young and aged samples (Figure S4F–K, Supporting Information). Given that over 90% of total thymocytes are CD4⁺CD8⁺ DP cells that have already undergone TCRβ recombination, both young and aged thymocytes exhibited higher VβDβJβ rearrangement frequencies than DN3 thymocytes, approaching the levels observed in pre‐selection DP thymocytes.^[^ ^35^ ^]^ This pattern is consistent with the developmental timing of *Tcrb* rearrangement.

In contrast, age‐related changes were more apparent in the TCRα repertoire. HTGTS‐TCR‐seq using proximal Jα bait primers (*Traj61*, *Traj56*, and *Traj45*) showed that aged thymocytes exhibited a reduced usage of Vα segments across the central repeat to distal unique *Trav* regions (Figure 3D, E; Figure S4L, Supporting Information). Notably, aged thymocytes also displayed increased usage frequencies of certain Vα segments within the proximal repeat region, particularly with *Traj56* and *Traj45* baits, whereas the usage of some individual Vα segments was decreased. When distal Jα bait primers (*Traj18* and *Traj9*) were used, a reduction in the usage of Vα segments from the proximal repeat region was observed in aged thymocytes, while several Vα segments from the central repeat to distal *Trav* regions showed increased usage frequencies (Figure 3F; Figure S4M, Supporting Information). To further characterize Jα segment usage, we applied four different Vα bait primers targeting either distal (*Trav2* and *Trav1*) or proximal (*Trav20* and *Trav17*) Vα segments. HTGTS‐TCR‐seq using distal Vα baits revealed a modest preferential usage of distal Jα segments in aged thymocytes; in contrast, Jα usage patterns with proximal Vα baits were largely comparable between young and aged mice (Figure 3G,H; Figure S4N,O, Supporting Information). Together, these results indicate that the major alterations in the aged thymic TCR repertoire reside in the TCRα chain.

### 2.6 Comparison of TCR Repertoires Between Peripheral CD4^+^ and CD8^+^ T Cells

**Figure 4:**
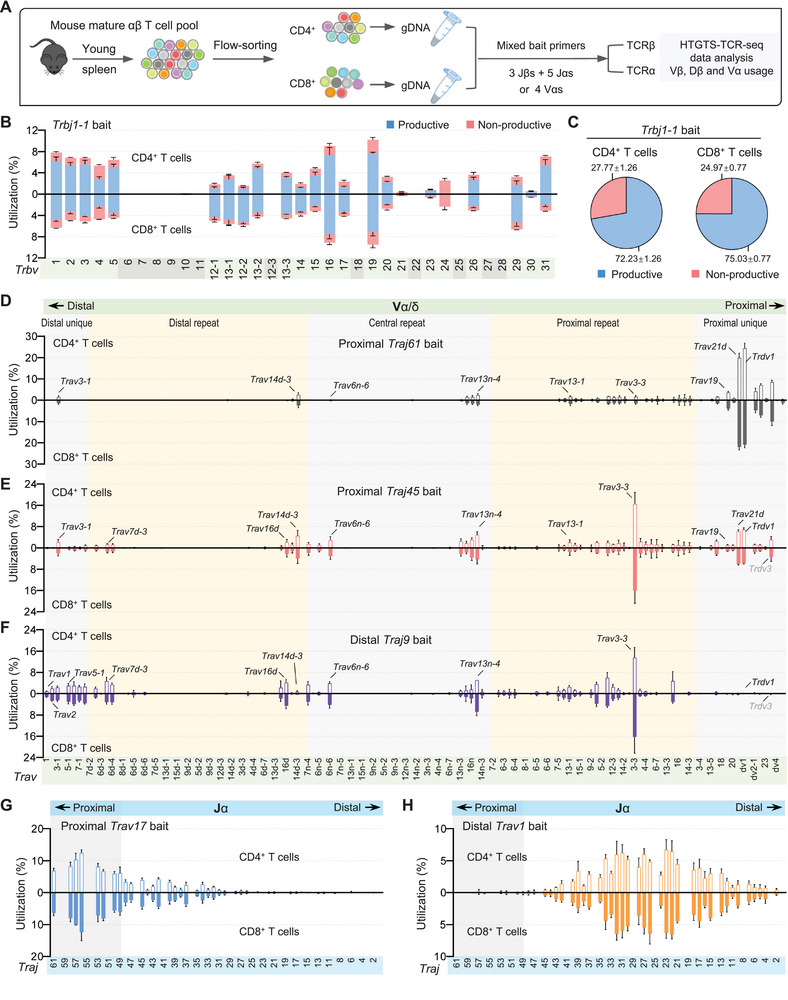
Comparison analysis of TCR repertoires in mature CD4^+^ and CD8^+^ T cells isolated from the spleens of young C57BL/6 mice. A) Schematic overview of the HTGTS‐TCR‐seq experimental workflow employed to profile TCRβ and TCRα repertoires in mature αβ T cells from young C57BL/6 mice. gDNA was extracted from sorted CD4^+^ and CD8^+^ T cells and subsequently employed with indicated mixed bait primers for library preparation. B) Vβ repertoire analysis with productive and nonproductive information from VβDβJβ rearrangements in CD4^+^ (up) and CD8^+^ (down) T cells using Jβ bait primer *Trbj1‐1*. Pseudogenes are denoted with gray background shading. C) Pie charts illustrating the average overall percentage of productive versus nonproductive VβDβJβ rearrangements in CD4^+^ (left) and CD8^+^ (right) T cells, as derived from the libraries shown in panels (B). D–F) Vα segment usage in VαJα rearrangements from mature T cells, with analysis conducted separately in CD4^+^ (up) and CD8^+^ (down) T cells using Jα bait primers *Traj61* (D), *Traj45* (E), and *Traj9* (F). For comparison, several Vα segments are highlighted. G,H) Jα segment usage in VαJα rearrangements in CD4^+^ (up) and CD8^+^ (down) T cells using Vα bait primers *Trav20* (G) and *Trav2* (H). Data are presented as mean ± s.d.; *n*, number of mice. See Table S2 and Figure S5–S7 (Supporting Information) for more information.

As most thymocytes are immature and have not undergone positive or negative selection, we next sought to characterize differences in the mature TCR repertoire between peripheral CD4⁺ and CD8⁺ T cells. This provided an ideal opportunity to evaluate whether HTGTS‐TCR‐seq can simultaneously assess V or J segment usage in both TCRα and TCRβ chains. To this end, we first sorted splenic CD4⁺ and CD8⁺ T cells from young C57BL/6 mice and generated HTGTS‐TCR‐seq libraries using mixed J and/or V primer pools (**Figure** **4**A).

For the TCRβ chain, peripheral CD4⁺ and CD8⁺ T cells displayed markedly different Vβ usage patterns compared to the overall thymic TCRβ repertoire from young mice analyzed using the same Jβ baits (Figure S5A, Supporting Information). For instance, via the *Trbj1‐1* bait, *Trbv2*, *Trbv3*, *Trbv5*, *Trbv15*, and *Trbv31* were more frequently used in CD4⁺ T cells, whereas *Trbv12‐1*, *Trbv13‐1*, *Trbv12‐2*, *Trbv13‐3*, *Trbv14*, *Trbv17*, and *Trbv29* were preferentially used in CD8⁺ T cells (Figure 4B; Figure S5B,C, Supporting Information). Notably, among the observed biases, only *Trbv31* showed consistently enrichment across all three *Trbj* bait primers. Nonproductive rearrangements were relatively rare, although *Trbv24* was predominantly involved in nonproductive VβDβJβ rearrangements in both subsets. Interestingly, we observed a higher proportion of nonproductive rearrangements in CD4⁺ T cells than in CD8⁺ T cells across all bait conditions (Figure 4C; Figure S5D,E, Supporting Information). Despite these differences, overall Dβ usage and recombination frequencies were largely comparable between CD4⁺ and CD8⁺ T cells (Figure S5F–K, Supporting Information).

In contrast, for the TCRα chain, Vα usage patterns were largely similar between peripheral CD4⁺ and CD8⁺ T cells, with only several Vα segments displaying subset‐specific enrichment. Nevertheless, the Vα repertoires of both peripheral subsets differed substantially from the thymic TCRα repertoire in young mice under the same Jα bait conditions. As Jα baits shifted from proximal (e.g., *Traj61*) to more distal positions (e.g., *Traj9*), Vα usage correspondingly shifted toward more distal Vα segments (Figure 4D–F; Figure S5L,M, Supporting Information). A similar positional bias was observed when using Vα bait primers to interrogate Jα usage: proximal Vα segments (e.g., *Trav20*, *Trav17*) preferentially recombined with proximal Jαs, while distal Vαs (e.g., *Trav2*, *Trav1*) favored distal Jα segments (Figure 4G, H; Figure S5N,O, Supporting Information). Consistent with the similarity in Vα usage, Jα usage also did not significantly differ between peripheral CD4⁺ and CD8⁺ T cells from young mice.

We next asked whether alterations in TCR repertoire could also be detected in aged peripheral T cells (Figure S6A, Supporting Information). Although Vβ usage patterns in aged CD4⁺ and CD8⁺ T cells largely resembled those of young mice, aged splenic T cells exhibited increased nonproductive rearrangements across all three Jβ baits, mirroring the high nonproductive joining rates seen in both young and aged thymocytes (Figure S6B–M, Supporting Information). Moreover, the overall V(D)J recombination frequency was reduced in aged mature T cells, particularly within the CD8⁺ subset (Figure S6N,O). While Vα usage did not significantly differ between aged CD4⁺ and CD8⁺ subsets, the constrained Vα repertoire patterns closely resembled those observed in aged thymocytes (Figure S7, Supporting Information). Given that mouse T cell maturation involves stringent positive and negative selection after TCR recombination in the thymus, the comparable features of TCR repertoire in aged peripheral T cells and thymocytes suggest that impaired thymic function may underlie these repertoire alterations.

Together, these results demonstrate that the primary differences in the young peripheral TCR repertoire between CD4⁺ and CD8⁺ T cells reside in the TCRβ chain, whereas, in the aged, repertoire alterations are detected in both TCRα and TCRβ chains, highlighting the capacity of HTGTS‐TCR‐seq to simultaneously and reproducibly profile V or J segment usage across both *Tcra* and *Tcrb* loci in a single assay.

### 2.7 Altered *Tcra* Gene Rearrangements upon WAPL Depletion Revealed by HTGTS‐TCR‐Seq

**Figure 5:**
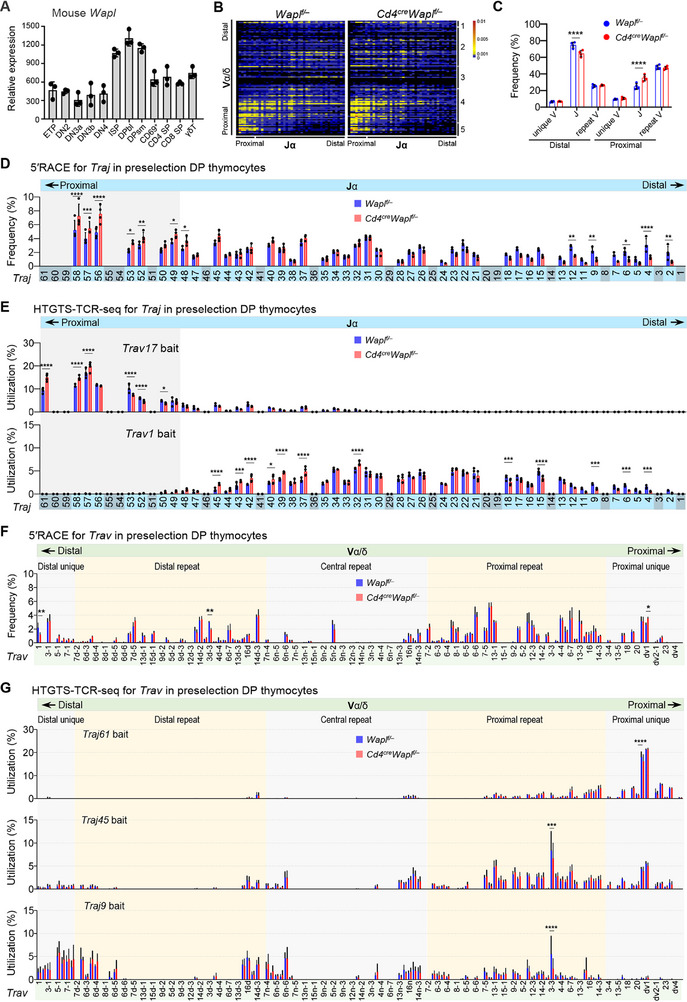
*Tcra* rearrangement in WAPL‐deficient preselection DP thymocytes. A) Relative *Wapl* expression across developing mouse T cells (ImmGen database). Data for the indicated cell types are shown. B) Representative heatmap of Vα‐Jα combination frequencies by 5′RACE in *Wapl^f/−^*, and *Cd4^cre^Wapl^f/−^* preselection DP thymocytes. 1, distal unique; 2, distal repeat; 3, central repeat; 4, proximal repeat; 5, proximal unique. Data are representative of three independent experiments. C,D) Grouped Vα–Jα combination frequencies (C) and relative Jα usage (D) from 5′RACE in *Cd4^cre^Wapl^f/−^* and control preselection DP thymocytes, calculated from panel (B). Proximal Jα segments are denoted in gray. E) Jα usage quantified by HTGTS‐TCR‐seq with genomic DNA from preselection DP thymocytes of indicated genotypes, using *Trav17* and *Trav1* bait primers. Pseudogenes and gene segments with nonfunctional RSSs are marked with gray background shading (D‐E). F) Relative Vα usage from 5′RACE in preselection DP thymocytes of the indicated genotypes, calculated from panel (B). G) Vα usage quantified by HTGTS‐TCR‐seq with genomic DNA from preselection DP thymocytes of indicated genotypes, using *Traj61*, *Traj45*, and *Traj9* bait primers. Two‐way ANOVA followed by Tukey's multiple‐comparisons test was performed. All data are represented as the mean ± s.d. in bar graphs. See Tables S2 and S3 and Figure S8 (Supporting Information) for more information.

Previous studies in mouse pro‐B cells have shown that reduced WAPL expression prolongs cohesin residence time on chromatin, thereby extending loop extrusion, promoting *Igh* locus contraction, and enabling long‐range V_H_‐to‐DJ_H_ recombination.^[^ ^40^, ^41^ ^]^ In contrast, analysis of thymocyte subsets showed that *Wapl* mRNA is detectable throughout T cell development, peaks in DP thymocytes, and decreases at the single‐positive stages (**Figure** **5**A).^[^ ^42^ ^]^ This expression pattern provided an excellent system to test whether HTGTS‐TCR‐seq could be used to investigate how WAPL dosage influences TCR assembly during thymocyte differentiation. To avoid the lethality caused by WAPL loss in dividing cells, we conditionally deleted *Wapl* using *Cd4*‐Cre at the DP stage, when thymocytes exit the cell cycle and initiate *Tcra* recombination (Figure S8A, Supporting Information). Although efficient *Wapl* deletion in preselection DP thymocytes led to dysregulation of certain genes, overall thymocyte differentiation proceeded normally, indicating that WAPL loss at the DP stage does not appear to overtly disrupt thymocyte development (Figure S8B–G, Supporting Information).

We next examined the role of WAPL in shaping the thymic *Tcra* repertoire using sorted preselection DP thymocytes. To compare mRNA‐ and DNA‐level recombination outcomes, we employed both 5′RACE and HTGTS‐TCR‐seq. Both assays consistently revealed a biased preference for proximal Jα usage in WAPL‐deficient thymocytes (Figure 5B,C). Specifically, WAPL‐deficient preselection DP cells showed increased usage of proximal Jα segments—including the pseudogene Jα61, detected only by HTGTS‐TCR‐seq—accompanied by a concomitant reduction in distal Jα usage. This skewing was observed with both proximal and distal Vα baits (Figure 5D,E; Figure S8H,I, Supporting Information). In contrast, Vα usage remained comparable between WAPL‐deficient and control thymocytes, indicating that WAPL loss selectively restricts progressive distal Jα recombination while favoring proximal Jα recombination (Figure 5F,G; Figure S8J,K, Supporting Information).

Together, these findings establish that WAPL plays a critical role in regulating *Tcra* locus recombination, particularly Jα usage, to ensure a diverse *Tcra* repertoire during thymocyte development, and further establish HTGTS‐TCR‐seq as a useful approach for probing the DNA‐level mechanisms of TCR assembly during mouse lymphocyte development.

### 2.8 HTGTS‐TCR‐Seq Enables Profiling of Human TCR Diversity in Peripheral αβ T Cells

**Figure 6:**
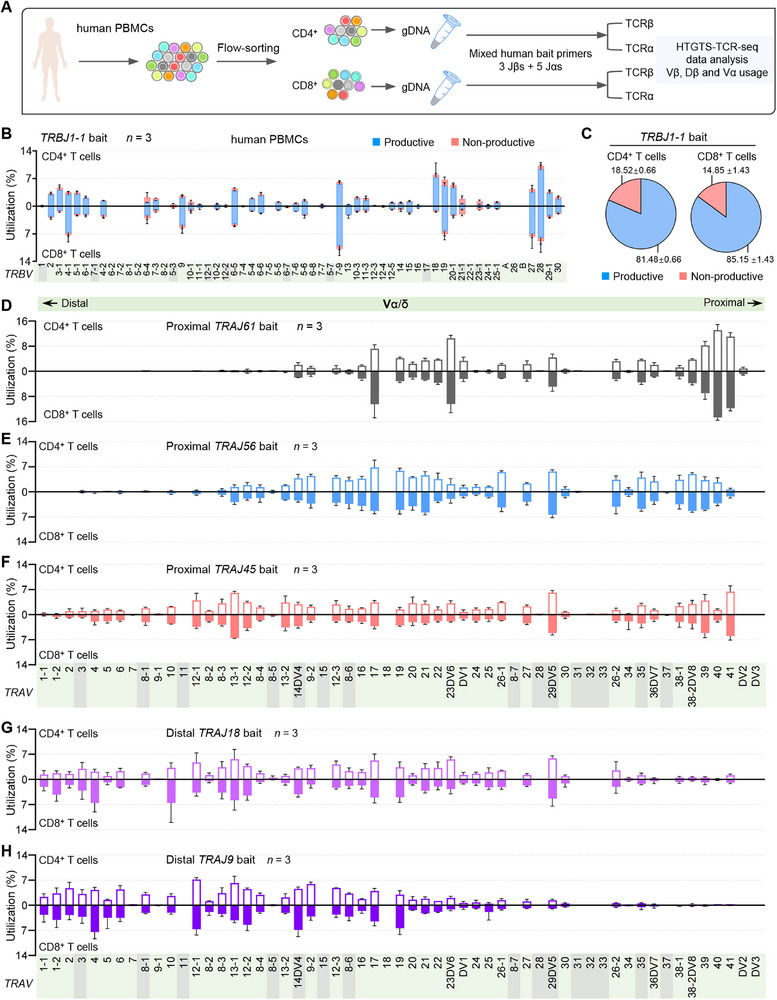
HTGTS‐TCR‐seq analysis of TCR repertoires in human mature αβ T cells. A) Schematic overview of the HTGTS‐TCR‐seq workflow for profiling TCRβ and TCRα repertoires in human CD4^+^ and CD8 T cells sorted from peripheral blood mononuclear cells (PBMCs). gDNA was extracted from CD4^+^ and CD8^+^ T cells sorted from human PBMCs and subsequently employed with indicated mixed J bait primers for library preparation. B) Analysis of the Vβ repertoire, displaying both productive and nonproductive VβDβJβ rearrangements in CD4^+^ (up) and CD8^+^ (down) T cells, using human Jβ bait primer *TRBJ1‐1*. Pseudogenes are denoted with gray background shading. C) Pie charts showing the average overall percentage of productive versus nonproductive VβDβJβ rearrangements from the libraries analyzed in panel (B) for CD4^+^ (left) and CD8^+^ (right) T cells. D–H) Average utilization frequencies of all Vα segments in VαJα rearrangements in CD4^+^ (up) and CD8^+^ (down) T cells, analyzed with proximal Jα bait primers *TRAJ61* (D), *TRAJ56* (E), and *TRAJ45* (F), and distal Jα bait primers *TRAJ18* (G) and *TRAJ9* (H). Pseudogenes are denoted with gray background shading. Data are presented as mean ± s.d.; *n*, number of donors. See Table S2 and Figure S9 (Supporting Information) for more information.

The human *TCRB* locus comprises 67 Vβ segments, of which 48 Vβs are functional. It includes two DβJβ clusters: each cluster contains a single Dβ segment (*TRBD1* or *TRBD2*), 7 Jβ segments (*TRBJ1‐1* to *1‐7* or *TRBJ2‐1* to *2‐7*), with *TRBJ1‐6* annotated as nonfunctional. The *TCRA* locus includes 54 Vα and 61 Jα segments, among which 8 Vα and 4 Jα segments are nonfunctional pseudogenes.^[^ ^43^ ^]^ Having established HTGTS‐TCR‐seq as a reliable method for profiling mouse TCR diversity, we extended its application to human T cells using an analogous approach. Specifically, we sorted CD4⁺ and CD8⁺ T cells from the peripheral blood of three healthy young adult donors and performed HTGTS‐TCR‐seq using human‐specific bait primers targeting *TRBJ* (*TRBJ1‐1*, *TRBJ2‐1*, and *TRBJ2‐7*) and *TRAJ* (*TRAJ61*, *TRAJ56*, *TRAJ45*, *TRAJ18*, and *TRAJ9*) segments (**Figure** **6**A; Figure S9A, Supporting Information). These bait segments were selected to mirror the spatial positioning and functional equivalency of murine bait primers.

For the TCRβ chain, HTGTS‐TCR‐seq revealed distinct and subset‐specific Vβ usage patterns between human CD4⁺ and CD8⁺ T cells, similar to prior observations in mice. For example, under the *TRBJ1‐1* bait primer, *TRBV3‐1*, *TRBV5‐1*, *TRBV6‐6*, *TRBV18*, and *TRBV20‐1* were preferentially used in CD4⁺ T cells, whereas *TRBV4‐1*, *TRBV9*, *TRBV7*‐9, *TRBV13*, and *TRBV27* showed higher usage in CD8⁺ T cells (Figure 6B). Notably, biased usage of *TRBV18*, *TRBV20*‐1, and *TRBV27* was consistently observed across all three *TRBJ* bait primers (Figure S9B,C, Supporting Information). Nonproductive rearrangements were relatively infrequent overall; however, *TRBV21‐1* and *TRBV23‐1* were predominantly involved in nonproductive events. A higher proportion of nonproductive rearrangements was detected in CD4⁺ T cells compared to CD8⁺ T cells when using the *TRBJ1‐1* and *TRBJ2‐1* baits, but no subset difference with the *TRBJ2‐7* bait (Figure 6C; Figure S9D,E, Supporting Information). Despite these differences, overall Dβ usage and recombination frequencies were largely comparable between CD4⁺ and CD8⁺ T cells (Figure S9F–K, Supporting Information). In contrast, TCRα repertoires exhibited highly similar Vα usage patterns between human CD4⁺ and CD8⁺ T cells. Similar to observations in mice, we also detected a stepwise rearrangement of Vα segments with different Jα baits. Rearrangement of proximal and middle Vα segments was observed with the most proximal nonfunctional *TRAJ61* and functional *TRAJ56* baits (Figure 6D,E). A broad distribution of Vα segments was detected with the proximal *TRAJ45* bait, while the distal *TRAJ18* and *TRAJ9* baits showed biased usage of distal and middle Vα segments (Figure 6F–H).

Taken together, these results demonstrate that HTGTS‐TCR‐seq can effectively and reproducibly capture the human TCR diversity. While *TCRB* repertoires exhibit clear subset‐specific features in CD4⁺ versus CD8⁺ T cells, *TCRA* Vα segment usage remains largely conserved between the two subsets. These findings suggest that divergence in TCRβ composition is a more prominent determinant of T cell subset identity in the human peripheral T cell pool.

### 2.9 HTGTS‐TCR‐Seq Reveals Conserved CDR3 Properties in Mouse and Human Peripheral αβ T Cells

As TCR repertoire diversity is primarily shaped by variations in the CDR3, particularly in the TCRβ chain, we next employed HTGTS‐TCR‐seq to profile the CDR3 features of productive VβDβJβ rearrangements in mouse and human peripheral αβ T cells.^[^ ^44^, ^45^ ^]^

In mouse splenic CD4⁺ and CD8⁺ T cells, CDR3 length distributions exhibited a unimodal pattern ranging from 8 to 16 amino acids, with a dominant peak at 11–12 residues across all three Jβ bait primers (Figure S10A–F, Supporting Information). Rearrangements involving *Trbj2‐1*, which encodes a longer J segment than *Trbj1‐1* or *Trbj2‐7*, yielded longer average CDR3 lengths (median 12 vs 11 aa). Comparable patterns were observed in human CD4⁺ and CD8⁺ T cells from PBMCs, where CDR3 lengths ranged from 5 to 20 amino acids with prominent peaks at 11–13 residues, again reflecting the influence of J segment length on CDR3 properties (Figure S10G–L, Supporting Information). Across both species, consensus CDR3 motifs of 11–13 amino acids were consistently recovered, with highly conserved residues at positions 1–3. These motifs were typically characterized by an N‐terminal alanine and a glutamate residue at the third‐to‐last position, independent of the specific Vβ segments. Notably, we identified a shared CDR3 motif between mouse and human peripheral T cells previously linked to self‐associated TCRs implicated in self‐renewal, autoimmunity, and tumor surveillance.^[^ ^46^, ^47^ ^]^ Together, these results demonstrate that HTGTS‐TCR‐seq reliably captures the structural features of productive VβDJβ rearrangements and reveal a conserved CDR3 architecture across species.

As proof of principle, we demonstrated that HTGTS‐TCR‐seq can capture human TCR diversity in peripheral αβ T cells. To evaluate its potential for clinical applications, we assessed the method's sensitivity using sorted splenic T cells from young mice. Libraries prepared from 2 and 0.1 µg of genomic DNA showed nearly identical Vβ repertoires (Figure S11A–F, Supporting Information), indicating that HTGTS‐TCR‐seq can reproducibly profile the TCR repertoire with as little as 0.1 µg of DNA. However, analysis of unique CDR3 sequences revealed that higher genomic DNA input increased both detection sensitivity and the number of overlapping CDR3s, suggesting that input DNA requirements may limit its potential applicability to clinical human samples (Figure S11G–L, Supporting Information).

## 3 Discussion

Our study demonstrates that HTGTS‐TCR‐seq, an application of HTGTS‐rep‐seq specifically adapted for αβ T cells, is an efficient, complementary, DNA‐based method for profiling TCRα and TCRβ repertoires with minimal primer bias. By utilizing linear amplification with a single or limited set of bait primers targeting J or V segments, this approach reduces the complexity and amplification bias associated with conventional multiplex PCR. Importantly, HTGTS‐TCR‐seq captures genomic rearrangements directly, enabling relative quantitative detection of both productive and nonproductive events independent of expression, which is a key advantage over RNA‐based methods.

To account for positional effects on recombination frequency, we designed HTGTS‐TCR‐seq bait primers across multiple genomic locations. This enabled us to resolve spatial patterns of TCR assembly and revealed locus‐specific principles. Vβ usage exhibited a consistent pattern across different baits, indicating that recombination is governed by stable locus‐wide features such as 3D chromatin topology, chromatin accessibility, and RSS quality.^[^ ^48^, ^49^, ^50^ ^]^ In contrast, the TCRα locus exhibited a stepwise rearrangement pattern, from 3′ to 5′ Vα and 5′ to 3′ Jα, consistent with successive primary and secondary recombination events. These data highlight HTGTS‐TCR‐seq as a useful tool for dissecting the spatial and temporal regulation of TCR α and β gene rearrangements and diversity at high resolution.

Our analysis of age‐associated changes in the thymic and peripheral TCR repertoire revealed an obvious divergence between TCR α and β gene rearrangements. While Vβ segment usage remained relatively stable with age, nonproductive joining rates increased, and TCRα repertoire diversity declined during age‐related thymic involution. Notably, constrained Vα repertoire patterns in aged peripheral T cells closely resembled those observed in aged thymocytes.^[^ ^38^, ^51^ ^]^ Given that naïve T cell production in mice depends almost entirely on thymic output throughout life, these alterations suggest a bottleneck in αβ chain pairing and thymic selection, ultimately restricting peripheral TCR diversity.^[^ ^52^, ^53^ ^]^ We further identified the cohesin unloader WAPL as a critical regulator of *Tcra* recombination during thymocyte differentiation. In the future, combining HTGTS‐TCR‐seq with 3C‐HTGTS could provide mechanistic insight into how dynamic chromatin loop extrusion regulates *Tcra* and *Tcrb* locus rearrangements. We also observed distinct Vβ usage patterns between peripheral CD4⁺ and CD8⁺ T cells, indicative of differential selection pressures shaped by MHC class II and class I environments, respectively.^[^ ^54^, ^55^, ^56^ ^]^ Despite these differences, both subsets retained comparable CDR3 length distributions and conserved core motifs, indicating that thymic selection imposes structural constraints on junctional diversity.^[^ ^55^ ^]^ In our validation assay, a higher amount of input DNA improved the quality and unique CDR3 detection. Together with the successful application of HTGTS‐TCR‐seq to human PBMC‐derived αβ T cells, these results underscore the utility of this method for profiling TCR α and β repertoires across developmental stages, aging, and limited clinical samples.

Several technical limitations should be noted. First, genomic DNA must be fragmented to limit LAM‐PCR product length, but sonication can partially disrupt target sequences. Moreover, capturing a highly diverse TCR CDR3 repertoire requires relatively large amounts of input DNA, which greatly restricts the method's applicability to rare clinical samples. Second, bait primers placed in close proximity can generate overlapping PCR products and interfere with one another. Highly similar or repetitive segments across TCR α and β gene loci cannot be targeted with specific primers, further limiting the number of usable baits and reducing representation of all possible rearrangement events. Third, differences in DNA template length and bait location can affect amplification efficiency. LAM‐PCR substantially reduces but cannot fully eliminate this bias. For absolute quantification, as in PEM‐seq, a substantially larger amount of input genomic DNA is required, along with incorporation of unique molecular identifiers (UMIs) in adaptor sequences and a UMI‐based data analysis pipeline.^[^ ^57^, ^58^ ^]^ Fourth, because *Tcra* and *Tcrb* gene rearrangements generate excision circles that persist in cells, our current HTGTS‐TCR‐seq does not distinguish rearrangements occurring on genomic DNA from those captured on excision circles, which may contribute to discrepancies with mRNA‐based approaches.^[^ ^59^ ^]^ Fifth, the high similarity among TCRα sequences in the C57BL/6 strain hampers the distinction of certain Vα segments, thereby making CDR3 analysis difficult. This limitation is less pronounced in 129/Sv mice and in humans. Finally, HTGTS‐TCR‐seq is currently optimized for αβ T cells using a limited set of bait primers and does not capture γδ T cell repertoires. Addressing these challenges will require expanded primer design, larger bait pools, and further optimization to enable truly comprehensive repertoire coverage across T cell subsets.

Despite these caveats, HTGTS‐TCR‐seq provides an efficient, complementary, and cost‐efficient approach for relative quantification of TCR repertoires at the DNA level. Compared with traditional approaches, it minimizes primer bias, directly captures genomic rearrangements including pseudogene usage, and enables resolution of the spatial and temporal dynamics of recombination. These features make HTGTS‐TCR‐seq a valuable tool for advancing our understanding of T cell immunity and the mechanisms governing TCR assembly, with considerable potential for applications in both basic and translational immunology.

## 4 Experimental Section

### Experimental Procedures

No statistical methods were used to predetermine sample size. Experiments on mice were not randomized, and investigators were not blinded to allocation during experiments and outcome assessment.

### Mice

Wild‐type 4‐ to 6‐week‐old (young) and 16‐ to 18‐month‐old (aged) C57BL/6 mice were maintained under specific pathogen‐free conditions on a 12 light/12 dark cycle in a temperature‐controlled environment, with food and water provided ad libitum. All animal experiments were performed under protocols (SIBCB‐S648‐2110‐034) approved by the Institutional Animal Care and Use Committee of the Institute of Biochemistry and Cell Biology, Center for Excellence in Molecular Cell Science, Chinese Academy of Sciences, China.

*Cd4*‐Cre transgenic mice were obtained from Z. Hua (Nanjing University). Conditional *Wapl* floxed mice were generated by introducing two loxP sites flanking exons 3 and 4 of the *Wapl* locus via homologous recombination in zygotes. For genome editing, sgRNA, Cas9 mRNA, and single‐stranded oligodeoxynucleotides (ssODN) were co‐injected into zygotes (C57BL/6×DBA) with the ssODNs serving as a template for homologous recombination. Using this strategy, mice carrying either the *Wapl^f/+^* or *Wapl^+/−^* genotype were generated. The *Wapl* floxed allele originated from the DBA background, whereas the *Wapl* germline knockout allele was derived from the C57BL/6 background. Because both the *Tcra* and *Wapl* loci are located on chromosome 14 with a relatively short intergenic distance, it was not feasible to analyze the *Tcra* locus in a pure C57BL/6 background for the conditional *Wapl^f/−^* genotype. Therefore, *Wapl^f/−^* mice with or without *Cd4*‐Cre on a mixed C57BL/6 and DBA background were generated by crossing *Wapl^f/+^* with *Wapl^+/−^* mice for subsequent experiments. In these mice, one *Tcra* allele originated from the C57BL/6 background and the other from the DBA background. Mice were maintained on a mixed C57BL/6 and DBA background and were age‐matched in all experiments. Sequences of primers and sgRNAs are listed in Table S1 (Supporting Information).

### T‐Cell Isolation from Mouse Thymus and Spleen

Single‐cell suspensions were prepared from thymuses and spleens by mechanical disruption with dissociation and filtration through a 70 µm nylon cell strainer. Red blood cells were lysed, and the remaining cells were pelleted and resuspended in phosphate‐buffered saline (PBS) with 2% FBS.

### Flow Cytometry and Cell Sorting

For isolation of DN3 T cells from young mice, CD4^+^ and CD8^+^ thymocytes were depleted by CD4/CD8 (TIL) MicroBeads (130‐116‐480, Miltenyi). The flow‐through was subsequently stained with PE‐anti‐CD25 (PC61, Biolegend, 102007), FITC‐anti‐CD44 (IM7, Biolegend, 103006), and Fixable Viability Stain 510 (BD Pharmingen, 564406). DN3 T cells were defined as CD4^−^CD8^−^CD44^−^CD25^+^. To isolate preselection DP thymocytes from young mice, total thymocytes were stained with APC‐Cy7‐anti‐CD4 (GK1.5, Biolegend, 100414), PerCP/Cyanine5.5‐anti‐CD8 (53‐6.7, Biolegend, 100734), PE‐Cy7‐anti‐CD69 (H1.2F3, Thermofisher‐Invitrogen, 25‐0691‐82), FITC‐anti‐TCRβ (H57‐597, Biolegend, 109205), and Fixable Viability Stain 510. Preselection DP T cell was defined as CD4^+^CD8^+^CD69^−^TCRβ^lo^. For isolation of splenic CD4^+^ or CD8^+^ T cells, splenic single‐cell suspensions were stained with APC‐Cy7‐anti‐CD4 (GK1.5, Biolegend, 100414), PerCP/Cyanine5.5‐anti‐CD8 (53‐6.7, Biolegend, 100734), and Fixable Viability Stain 510. Cell sorting was performed on FACSAria III or FACSAria Fusion instruments (BD) to isolate specific cell populations for further analysis.

### Isolation of Human T Cells from Peripheral Blood Mononuclear Cells

Peripheral blood from healthy donors was collected with institutional approval and informed consent. All human sample study was performed under the Institutional guidelines of CEMCS (2025‐111). Whole blood was diluted 1:1 with PBS, layered over Ficoll‐Paque PLUS (17144002‐1, Cytiva), and centrifuged for gradient density separation. The PBMC layer was collected, washed with PBS, counted using a hematocytometer, aliquoted, and cryopreserved in 90% FBS/10% dimethyl sulfoxide (DMSO) before storage in liquid nitrogen.

Frozen human PBMCs were thawed at 37 °C, resuspended in PBS with 2% FBS, and stained with PE‐anti‐human CD8a (HIT8a, Biolegend, 300908), APC‐anti‐human CD4 (RPA‐T4, Biolegend, 300552), FITC‐anti‐human CD19 (SJ25C1, Biolegend, 363008), and Fixable Viability Stain 510. CD4^+^ and CD8^+^ T cells were isolated by FACS using FACSAria III or FACSAria Fusion instruments (BD).

### Primer Design for HTGTS‐TCR‐Seq

Bio‐primers were designed within 20–200 bp downstream of the target sequences, such as J or V segments. Nested primers were positioned between the target segment and the bio‐primer, at a distance of <70 bp from the target sequence, and could partly overlap with target segments while avoiding dimer and hairpin formation. Primers were designed with annealing temperatures of 59–60 °C and GC contents of 40–50% (not exceeding 60%). Baits were selected from either functional or pseudogene segments that had been validated to undergo rearrangement by 5′RACE or multiplex PCR. To prevent overlap and interference among primers in the densely organized Jβ regions, mouse Jβ1‐1, Jβ1‐3, Jβ1‐4, Jβ2‐1, and Jβ2‐7 segments were chosen for bait design, with bio‐primers spaced at least 445 bp apart. Primer interference and capture efficiency were assessed both individually and in mixtures, with comparable performance observed in both conditions, confirming the absence of mutual interference. The final primer set for mouse and human included Jβ1‐1, Jβ2‐1, and Jβ2‐7, representing different regions of the locus, with bio‐primers spaced over 1000 bp apart. For the *Tcra* locus, bait primers were designed at frequently used Jα or Vα segments spanning proximal to distal regions, avoiding highly similar or repetitive segments, guided by published 5′RACE or quantitative PCR datasets. Baits for pseudogene segments involved in rearrangements were also included to comprehensively capture the rearrangement events. Sequences of all oligos used are listed in Table S1 (Supporting Information).

### HTGTS‐TCR‐Seq and Data Analysis

HTGTS‐TCR‐seq was performed as described with minor modifications.^[^ ^24^ ^]^ Genomic DNA was extracted from sorted DN3 or preselection T cells, total thymocytes, sorted splenic T cells, or human peripheral blood T cells. Briefly, 0.1–20 µg DNA was fragmented via sonication on Qsonic bioruptor and subjected to linear PCR amplification with a biotinylated primer as follows: PCR conditions: 95 °C 2 min; 95 °C 30 s; 58 °C 30 s; 72 °C 90 s; 80 cycles; 72 °C 2 min. Primers were removed by VAHTS DNA Clean Beads (Vazyme, N411). The resulting single‐strand biotinylated PCR products were collected via Dynabeads MyONE C1 streptavidin beads (ThermoFisher, 65 002) and ligated to bridge adapters overnight in the presence of 15% PRG8000. Ligation was conducted following 25 °C 60 min; 22 °C 120 min; 16 °C 9–12 h. Subsequently, i5‐indexed bait‐specific inner primers and i7‐index adapter primers were added to adapter‐ligated products by nested PCR. Excess primers were removed by VAHTS DNA Clean Beads, and the purified PCR products were further tagged with Illumina sequencing adapter sequences. Libraries were size‐selected by resolving PCR products on a 2% agarose gel, isolating fragments between 300 and 700 bp for mouse or human samples, with recovery performed via gel extraction. The HTGTS‐TCR‐seq libraries were sequenced on Novaseq X plus (Illumina) using 150‐bp paired‐end reads. Primer sequences are listed in Table S1 (Supporting Information).

For *Tcra* and *Tcrb* gene rearrangement analysis, sequencing reads were aligned to the mm10 reference genome for mouse T cells and to the hg38 reference genome for human T cells. All duplicate junctions were retained in the analyses for the reasons previously described.^[^ ^24^ ^]^ To quantify the utilization level of V, D, and J segments in TCR α and β gene rearrangements, we defined “on‐target” sequences as the 40‐bp regions flanking the recombination signal sequences (RSS) (RSS ± 40 bp), which were then used to determine the usage of V(D)J segments. To quantify TCR repertoires, we employed a pipeline called “HTGTSrep” to identify the productive and nonproductive V(D)J rearrangements, as well as to analyze the motifs and length distribution of CDR3 regions.^[^ ^22^ ^]^ *Trav3‐3* and *Trav3d‐3* are not distinguishable; both were maintained in this analysis, but the computed distribution of reads between the segments should be ignored.

### 5′RACE and Data Analysis

5′RACE was performed on total thymocytes as described. Briefly, Single‐cell suspensions were counted, lysed with TRIzol (15596018CN, Thermofisher‐Invitrogen), and total RNA was extracted. RNA (800 ng) was used for template switch with Superscript II (18064014, Thermofisher‐Invitrogen) with a template switch oligo (TSO). TCR amplification was performed using a universal forward primer and *Trac* or *Trbc*‐specific reverse primers. PCR products (300–700 bp) were gel‐purified. Libraries were prepared with the VAHTS Universal DNA Library Prep Kit for Illumina V3 (Vazyme, ND607‐01) and sequenced (2×150 bp paired‐end) on the Illumina Novaseq XPlus platform.

5′RACE data analysis was performed using MiXCR “analyze generic‐amplicon” function(v4.4.1).^[^ ^60^ ^]^ And then, the VDJtools (v1.2.1) command PlotFancyVJUsage was used to calculate clonal frequencies of V–J recombination.^[^ ^61^ ^]^ Heatmaps were generated using the pheatmap and RColorBrewer packages. *Trav3‐3* and *Trav3d‐3* are not distinguishable; both were maintained in this analysis, but the computed distribution of reads between the segments should be ignored.

### Bulk RNA‐Seq and Data Analysis

Total RNA was extracted with the TRIzol (15596018CN, ThermoFisher). Poly(A) mRNA was isolated using Oligo(dT) beads, fragmented with divalent cations at high temperature, and reverse‐transcribed with random primers. Double‐stranded cDNA was synthesized, end‐repaired, A‐tailed, and adaptor‐ligated. Adaptor‐ligated DNA was size‐selected with DNA Clean Beads and amplified with P5/P7 primers. Indexed libraries were validated, multiplexed, and sequenced on an Illumina NovaSeq 6000 (2×150 bp paired‐end).

For RNA‐seq analysis, the adaptor sequences were first trimmed from sequencing reads using Cutadapt (v4.1) with “‐e 0.1 ‐O 3 ‐m 55 ‐j 8 –quality‐cutoff 25” parameters.^[^ ^62^ ^]^ Trimmed reads were mapped to the mouse genome (mm10) with STAR (v2.5.2b).^[^ ^63^ ^]^ Genes with differential expression were detected using the Bioconductor DESeq2 package (v1.46.0) with adjusted *p* ≤ 0.05 and |log2 fold change | ≥ 1.^[^ ^64^ ^]^

### Quantification and Statistical Analysis

All experiments described above were performed at least three times. Statistical analyses were generated using GraphPad Prism 10.0 and R 3.6.3. Data was shown without pre‐processing. Statistics were presented as mean ± SD with sample size (n) at each figure. Unpaired two‐sided Student's *t*‐test, and two‐way ANOVA with Šídák post hoc test were used to calculate *p* values as follows: ^*^ *p* ≤ 0.05, ^**^ *p* ≤ 0.01, ^***^ *p* ≤ 0.001, and ^****^ *p* ≤ 0.0001.

## Conflict of Interest

Patent applications have been filed relating to the HTGTS‐TCR‐seq assay (Application number in China: CN202510466824.6).

## Author Contributions

R.L. and Y.S. contributed equally to this work. H.D. conceived the original idea, planned the project, and together with R.L., Y.S., Z.L., and W.W. designed the experiments, revised and approved the manuscript. R.L. performed most of the experiments; Y.S. performed most bioinformatic analyses; M.W. and L.Z. performed the molecular biological experiments and analyzed the data; R.L., L.Z., T.Y., and G.W. contributed to the TCR repertoire of human PBMCs. F.J. constructed mice. R.L., Y.S., W.W., and H.D. wrote the paper. H.D. supervised the study.

## Supporting information



Supporting Information

Supplemental Table 1

Supplemental Table 2

Supplemental Table 3

Supplemental Table 4

## Data Availability

The data that support the findings of this study are openly available in the Gene Expression Omnibus database at https://www.ncbi.nlm.nih.gov/geo/query/acc.cgi?acc=GSE308002, accession number GSE308002.
