# Supplementary material for: HTGTS‐TCR‐Seq for Profiling of Mouse and Human T‐Cell Receptor α and β Gene Rearrangements and Diversity
Source: Adv Sci (Weinh). 2025 Oct 27;13(4):e09497. doi: 10.1002/advs.202509497 (PMC12822386; doi:10.1002/advs.202509497)
Supplement: Supplementary file 1 — Supporting Information [file ADVS-13-e09497-s005.pdf]

Supporting Information

HTGTS-TCR-seq for Profiling of Mouse and Human T-cell Receptor α and β Gene Rearrangements and Diversity

Rui Luo, Yawei Song, Meichen Wang, Longhao Zou, Fangtai Jiao, Tiange Yang, Guangchuan Wang, Zhuoyi Liang, Wei Wu, Hai-Qiang Dai**

*Correspondence to: wuw@sibcb.ac.cn; haiqiang.dai@sibcb.ac.cn

Figure. S1

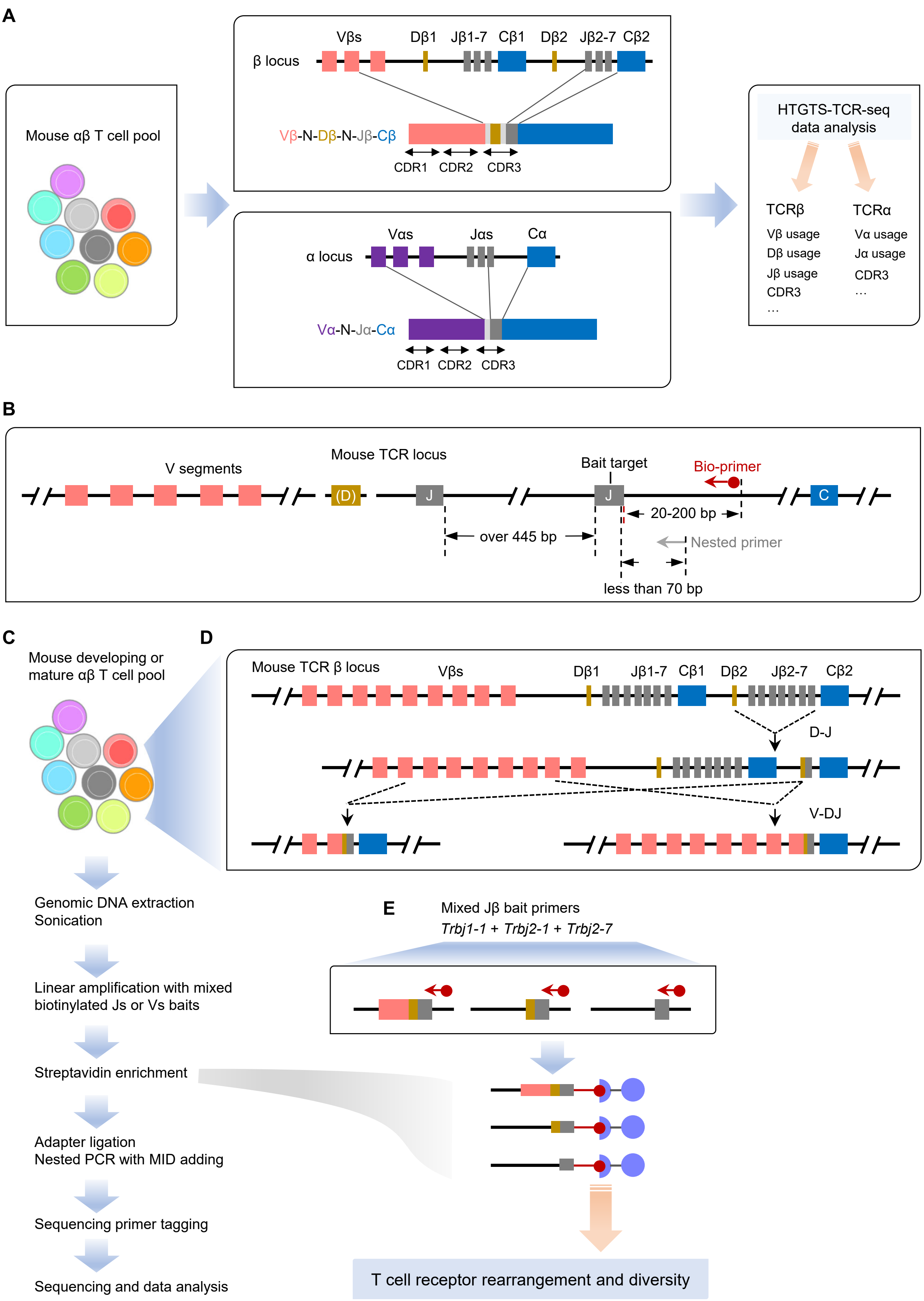
A
Mouse αβ T cell pool
Vβs
Dβ1
Jβ1-7
Cβ1
Dβ2
Jβ2-7
Cβ2
β locus
Vβ-N-Dβ-N-Jβ-Cβ
CDR1
CDR2
CDR3
Vαs
Jαs
Cα
α locus
Vα-N-Jα-Cα
CDR1
CDR2
CDR3
HTGTS-TCR-seq data analysis
TCRβ
Vβ usage
Dβ usage
Jβ usage
CDR3
…
TCRα
Vα usage
Jα usage
CDR3
…
B
Mouse TCR locus
V segments
(D)
J
Bait target
J
Bio-primer
C
20-200 bp
over 445 bp
Nested primer
less than 70 bp
C
Mouse developing or mature αβ T cell pool
Genomic DNA extraction
Sonication
Linear amplification with mixed biotinylated Js or Vs baits
Streptavidin enrichment
Adapter ligation
Nested PCR with MID adding
Sequencing primer tagging
Sequencing and data analysis
D
Mouse TCR β locus
Vβs
Dβ1
Jβ1-7
Cβ1
Dβ2
Jβ2-7
Cβ2
D-J
V-DJ
E
Mixed Jβ bait primers
Trbj1-1 + Trbj2-1 + Trbj2-7
T cell receptor rearrangement and diversity

Figure S1. HTGTS-TCR-Seq workflow for αβ T cell repertoire analysis. (**A**) Schematic representation illustrating the analysis of TCR α and β gene rearrangements and diversity via HTGTS-TCR-seq. Functional TCR-αβ chains are formed through the recombination of a variable (V), diversity (D), and joining (J) segment to a constant region (C) for the β-chain (middle, up panel), through V-J-to-C for the α-chain (middle, down panel). TCR diversity is further enhanced by addition and deletion of nucleotides (N) at the junctions between segments. CDR, complementarity-determining region. (**B**) Schematic illustrating the principle of primer design using a J-segment targeting sequence as an example. (**C**) Schematic showing the generation of DJβ and VβDJβ rearrangements via V(D)J recombination in developing T cells, focusing on the TCR β locus as a representative example of DJβ and VβDJβ joining events. (**D-E**) Overview HTGTS-TCR-seq method, using the TCR β chain as an example. Genomic DNA (gDNA) from T-cell populations is sonicated and subjected to linear amplification using the mixed biotinylated primers that anneal either downstream of specific Jβ-segments. The resulting biotin-labeled single-stranded DNA products are captured using streptavidin beads. Subsequent unbiased ligation of 3' ends with a bridge adaptor is performed. HTGTS-TCR-seq libraries are sequenced on Illumina NovaSeq X plus platforms with 150 bp paired-end reads. Sequence reads are processed and analyzed as described in Methods. MID, multiple identifier.

Figure. S2

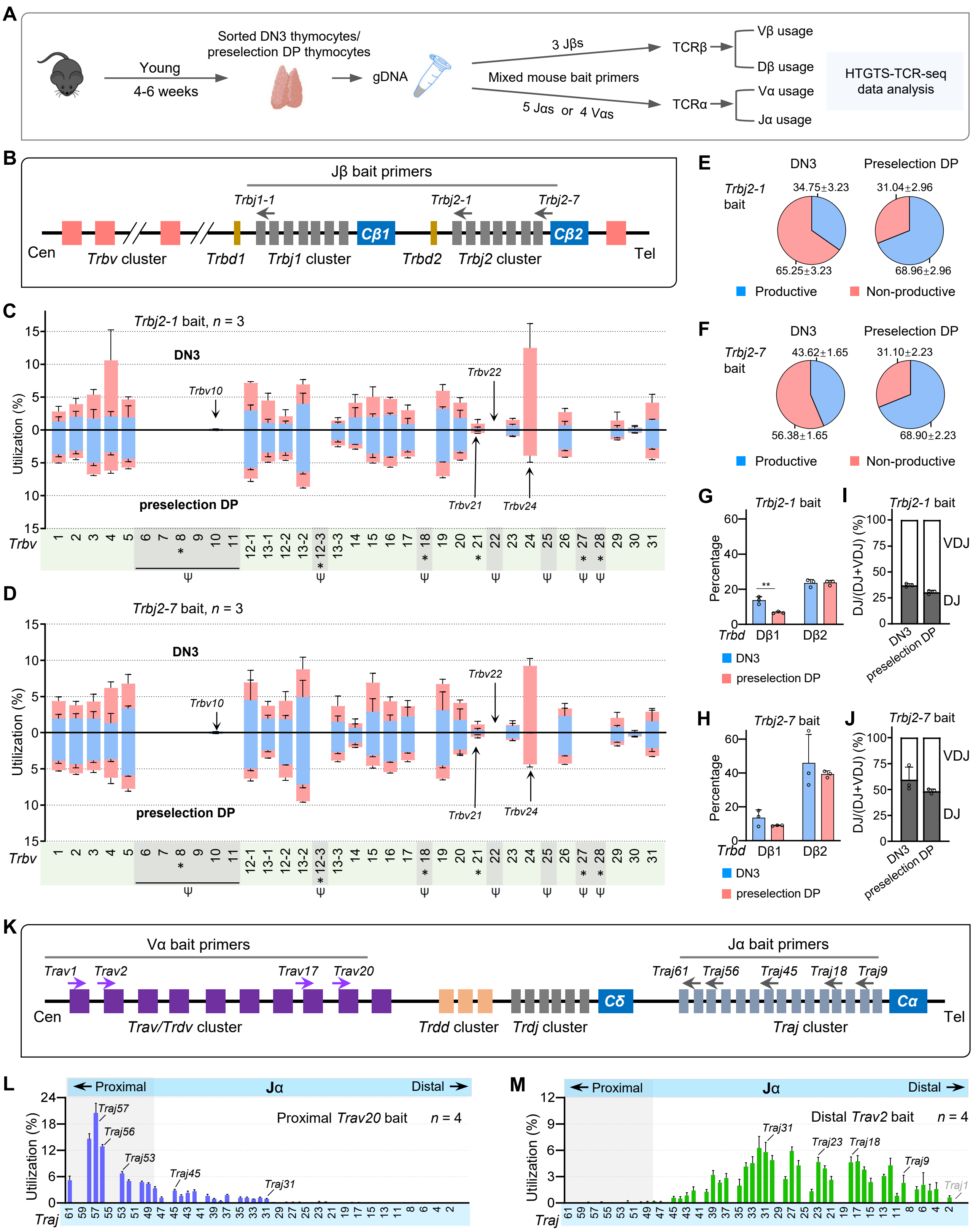

A
Young
4-6 weeks
Sorted DN3 thymocytes/ preselection DP thymocytes
gDNA
3 Jβs
Mixed mouse bait primers
5 Jαs or 4 Vαs
TCRβ
TCRα
Vβ usage
Dβ usage
Vα usage
Jα usage
HTGTS-TCR-seq data analysis
B
Jβ bait primers
Trbj1-1
Trbj2-1
Trbj2-7
Cβ1
Cβ2
Cen
Trbv cluster
Trbd1
Trbj1 cluster
Trbd2
Trbj2 cluster
Tel
C
Trbj2-1 bait, n = 3
DN3
preselection DP
Utilization (%)
Trbv10
Trbv22
Trbv21
Trbv24
Trbv
D
Trbj2-7 bait, n = 3
E
DN3
Preselection DP
Trbj2-1 bait
34.75±3.23
65.25±3.23
31.04±2.96
68.96±2.96
Productive
Non-productive
F
Trbj2-7 bait
43.62±1.65
56.38±1.65
31.10±2.23
68.90±2.23
G
Trbj2-1 bait
Percentage
Trbd
Dβ1
Dβ2
DN3
preselection DP
H
Trbj2-7 bait
I
J
DJ/(DJ+VDJ) (%)
VDJ
DJ
K
Vα bait primers
Jα bait primers
Trav1
Trav2
Trav17
Trav20
Traj61
Traj56
Traj45
Traj18
Traj9
Cδ
Cα
Cen
Trav/Trdv cluster
Trdd cluster
Trdj cluster
Traj cluster
Tel
L
Proximal
Jα
Distal
Proximal Trav20 bait
n = 4
Traj57
Traj56
Traj53
Traj45
Traj31
Utilization (%)
Traj
M
Distal Trav2 bait
Traj31
Traj23
Traj18
Traj9
Traj1

Figure S2. HTGTS-TCR-seq analysis of *Tcrb* gene rearrangement in DN3 thymocytes and preselection DP thymocytes, and *Tcra* gene rearrangement in sorted preselection DP thymocytes from young C57BL/6 mice (Related to Figure 1-2). (**A**) Overview of the HTGTS-TCR-seq experimental design for profiling the TCRβ repertoire in sorted DN3 and preselection DP thymocytes and TCRα repertoire in sorted preselection DP thymocytes. (**B**) Schematic representation of the murine *Tcrb* locus, with the locations of Jβ bait primers above the *Trbj* clusters depicted as gray arrows. (**C-D**) Vβ repertoire profiles with productive and nonproductive information from VβDβJβ rearrangements in DN3 (up) and preselection DP (down) thymocytes, using Jβ bait primers *Trbj2-1* (**C**) and *Trbj2-7* (**D**). Pseudogenes are denoted by ψ with grey background shading, and gene segments with nonfunctional RSSs are marked with an asterisk. (**E-F**) Pie charts showing the average overall percentage of productive versus nonproductive VβDβJβ rearrangements determined using bait primers *Trbj2-1* (**E**) and *Trbj2-7* (**F**) for DN3 (left) and preselection DP (right) thymocytes. Data in panels (**E-F**) correspond to the libraries analyzed in panels (**C-D**). (**G-H**) Comparison analysis of Dβ segment usage within DβJβ rearrangements in DN3 (blue) and preselection DP (red), as revealed with bait primers *Trbj2-1* (**G**) and *Trbj2-7* (**H**). *P* values were calculated via Student′s t-test; $^{*}P \leq 0.05$, $^{**}P \leq 0.01$ and $^{***}P \leq 0.001$. (**I-J**) Comparison of DβJβ: VβDβJβ ratios in sorted DN3 (left) and preselection DP (right), determined using bait primers *Trbj2-1* (**I**) and *Trbj2-7* (**J**). (**K**) Schematic representation of the murine *Tcra-Tcrd* locus. The positions of five Jα bait primers and four Vα bait primers are indicated above the *Tcra-Tcrd* clusters by gray and purple directional arrows, respectively. (**L-M**) Jα segment usage in VαJα rearrangements in sorted preselection DP thymocytes using proximal Vα bait primer *Trav20* (**L**) and distal Vα bait primer *Trav2* (**M**). For comparison, several Vα and Jα segments are indicated. Data are presented as mean ± s.d.; *n*, number of mice.

Figure. S3

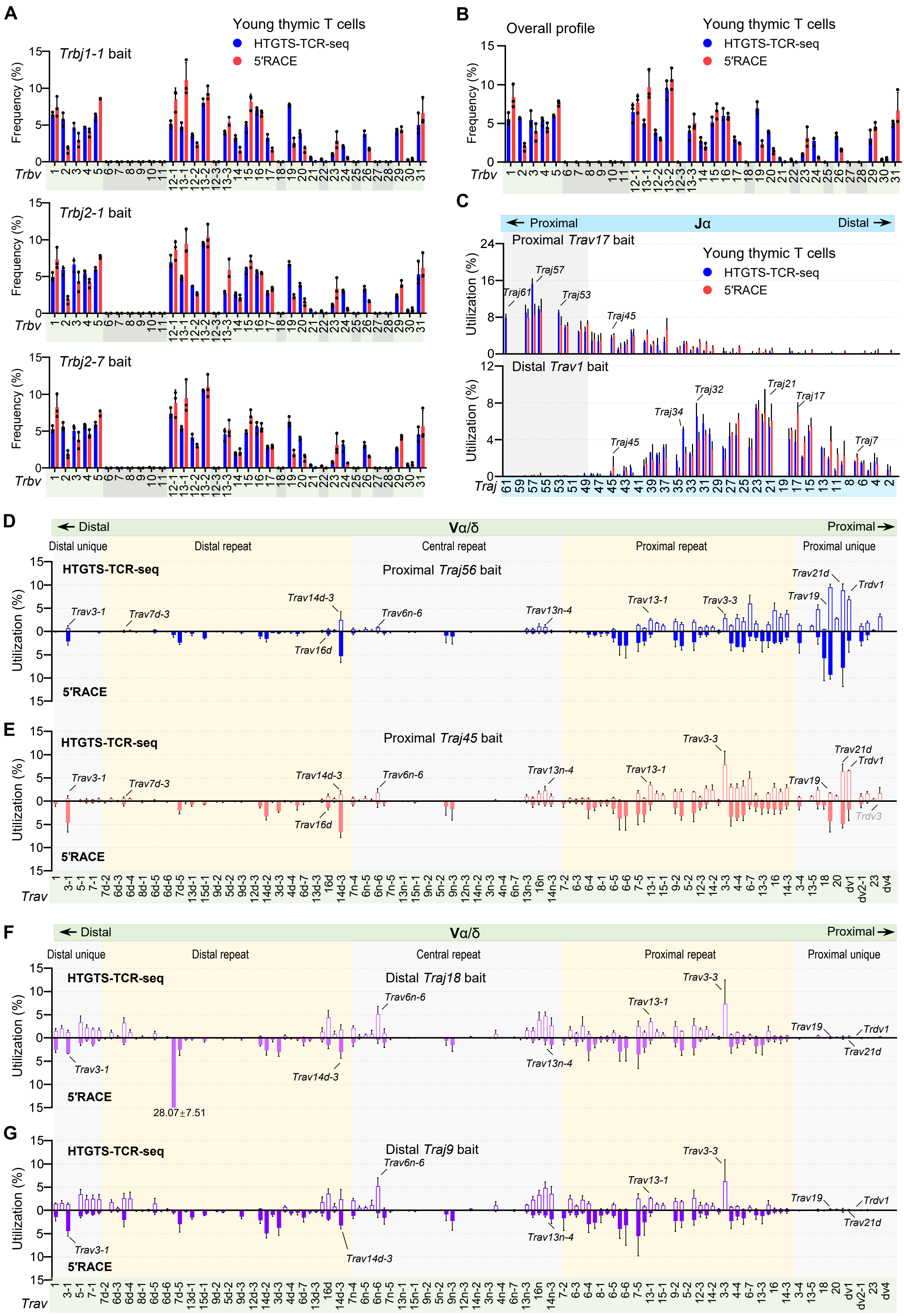
A
Young thymic T cells
HTGTS-TCR-seq
5′RACE
Trbj1-1 bait
Trbj2-1 bait
Trbj2-7 bait
Frequency (%)
Trbv
B
Overall profile
C
Proximal
Jα
Distal
Proximal Trav17 bait
Distal Trav1 bait
Utilization (%)
Traj
D
Distal
Vα/δ
Proximal
Distal unique
Distal repeat
Central repeat
Proximal repeat
Proximal unique
HTGTS-TCR-seq
Proximal Traj56 bait
5′RACE
E
Proximal Traj45 bait
Trav
F
Distal Traj18 bait
28.07±7.51
G
Distal Traj9 bait

Figure S3. Comparison of Vβ and Vα usage in young thymocytes using gDNA- vs. mRNA-based methods. (**A**) Vβ recombination frequencies determined by HTGTS-TCR-seq and 5′RACE using *Trbj1-1* (top), *Trbj2-1* (middle), and *Trbj2-7* (bottom) segmets. Libraries were generated from thymocytes. In HTGTS-TCR-seq, the frequency of each Vβ segment reflects its proportion of V(D)J rearrangements. (**B**) Overall Vβ recombination frequencies based on relative Vβ usage derived from HTGTS-TCR-seq and 5′RACE. HTGTS-TCR-seq data are presented as the mean across the three baits in (**A**). (**C**) Jα recombination frequencies determined by HTGTS-TCR-seq and 5′RACE using *Trav17* (top panel) and *Trav1* (bottom panel) baits. (**D-G**) Vα recombination frequencies determined by HTGTS-TCR-seq and 5′RACE using *Traj56* (**D**), *Traj45* (**E**), *Traj18* (**F**), and *Traj9* (**G**) segments. Data are presented as mean ± s.d.; HTGTS-TCR-seq (n = 3) and 5′RACE (n = 3) are shown. HTGTS-TCR-seq data are derived from Figure 3B and Figure S4 B-C. n, number of mice.

Figure. S4

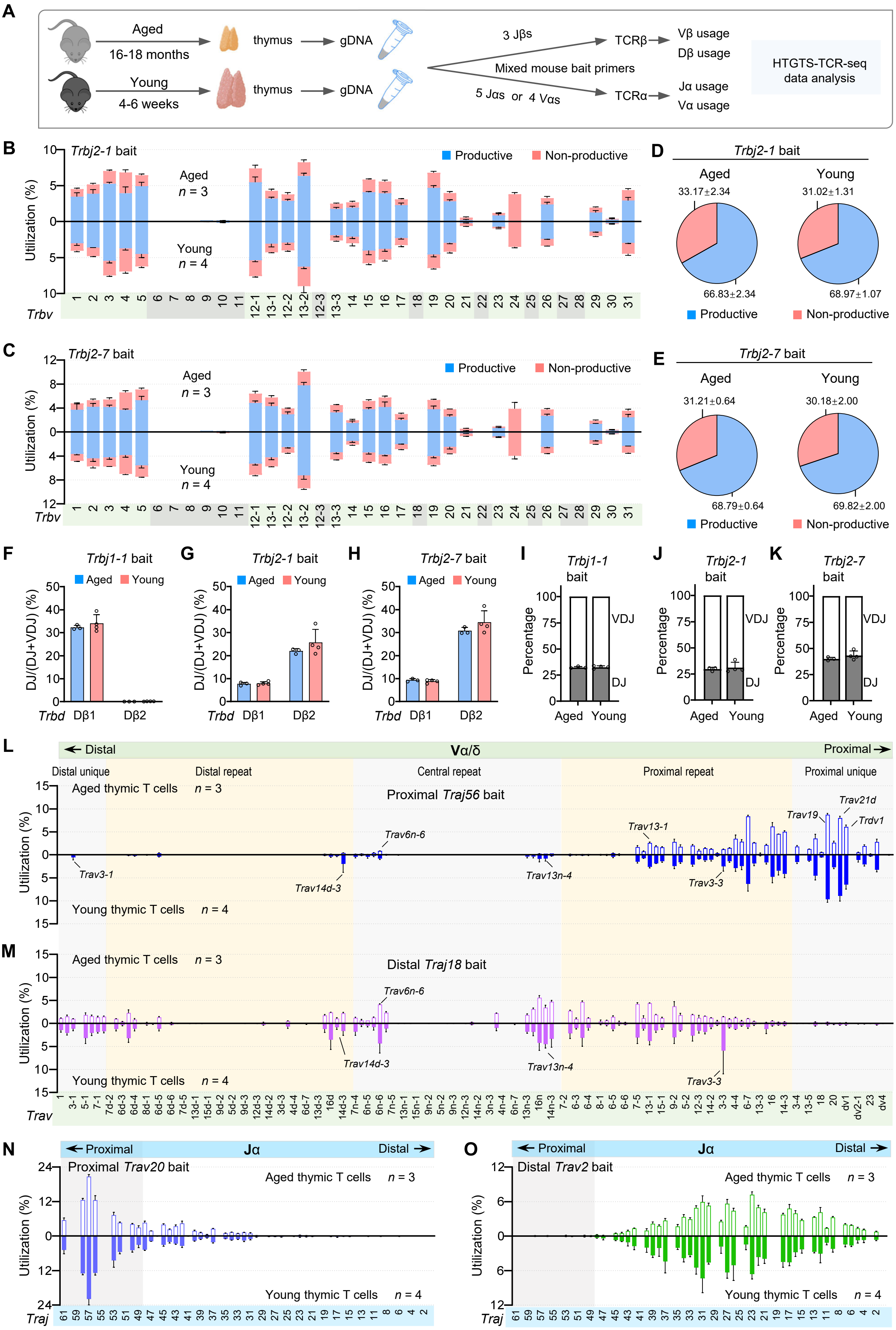

A
Aged
16-18 months
Young
4-6 weeks
thymus
gDNA
3 Jβs
Mixed mouse bait primers
5 Jαs or 4 Vαs
TCRβ
TCRα
Vβ usage
Dβ usage
Jα usage
Vα usage
HTGTS-TCR-seq data analysis
B
Trbj2-1 bait
Productive
Non-productive
Aged n = 3
Young n = 4
Utilization (%)
Trbv
C
Trbj2-7 bait
D
Trbj2-1 bait
Aged
Young
33.17±2.34
66.83±2.34
31.02±1.31
68.97±1.07
E
Trbj2-7 bait
31.21±0.64
68.79±0.64
30.18±2.00
69.82±2.00
F
Trbj1-1 bait
G
Trbj2-1 bait
H
Trbj2-7 bait
DJ/(DJ+VDJ) (%)
Trbd Dβ1 Dβ2
I
Trbj1-1 bait
J
Trbj2-1 bait
K
Trbj2-7 bait
Percentage
VDJ
DJ
L
Distal
Vα/δ
Proximal
Distal unique
Distal repeat
Central repeat
Proximal repeat
Proximal unique
Aged thymic T cells n = 3
Young thymic T cells n = 4
Proximal Traj56 bait
Trav3-1
Trav14d-3
Trav6n-6
Trav13n-4
Trav13-1
Trav3-3
Trav19
Trav21d
Trdv1
M
Distal Traj18 bait
Trav
N
Proximal
Jα
Distal
Proximal Trav20 bait
Traj
O
Distal Trav2 bait

Figure S4. Comparison analysis of TCR repertoires in aged and young thymocytes of C57BL/6 mice (Related to Figure 3). (**A**) Overview of the HTGTS-TCR-seq experimental design for profiling the TCRβ and TCRα repertoires with indicated bait primers in total thymocytes from aged and young C57BL/6 mice. (**B-C**) Vβ repertoire profiles with productive and nonproductive information from VβDJβ rearrangements in aged (up) and young (down) thymocytes, using Jβ bait primers *Trbj2-1* (**B**) and *Trbj2-7* (**C**). Pseudogenes are denoted with grey background shading. (**D-E**) Pie charts showing the average overall percentage of productive versus nonproductive VβDβJβ rearrangements derived from libraries shown in panel (**B-C**) for aged (left) and young (right) thymocytes. (**F-H**) Comparison analysis of Dβ segment usage within DJβ rearrangements in aged (blue) and young (red) thymocytes, as revealed with bait primers *Trbj1-1* (**F**), *Trbj2-1* (**G**), and *Trbj2-7* (**H**). (**I-K**) Comparison of DJβ:VβDJβ ratios in aged and young thymocytes, determined using bait primers *Trbj1-1* (**I**), *Trbj2-1* (**J**), and *Trbj2-7* (**K**). Data in panels (**F-K**) correspond to the libraries analyzed in panels (**B-C**) and Figure 3 panel (**B**). (**L-M**) Vα segment usage in VαJα rearrangements from aged (up) and young (down) thymocytes using Jα bait primers *Traj56* (**L**) and *Traj18* (**M**). For comparison, several Vα segments are highlighted. (**N-O**) Jα segment usage in VαJα rearrangements in aged (up) and young (down) thymocytes using Vα bait primer *Trav20* (**N**) and *Trav2* (**O**). Data are presented as mean ± s.d.; *n*, number of mice.

Figure. S5

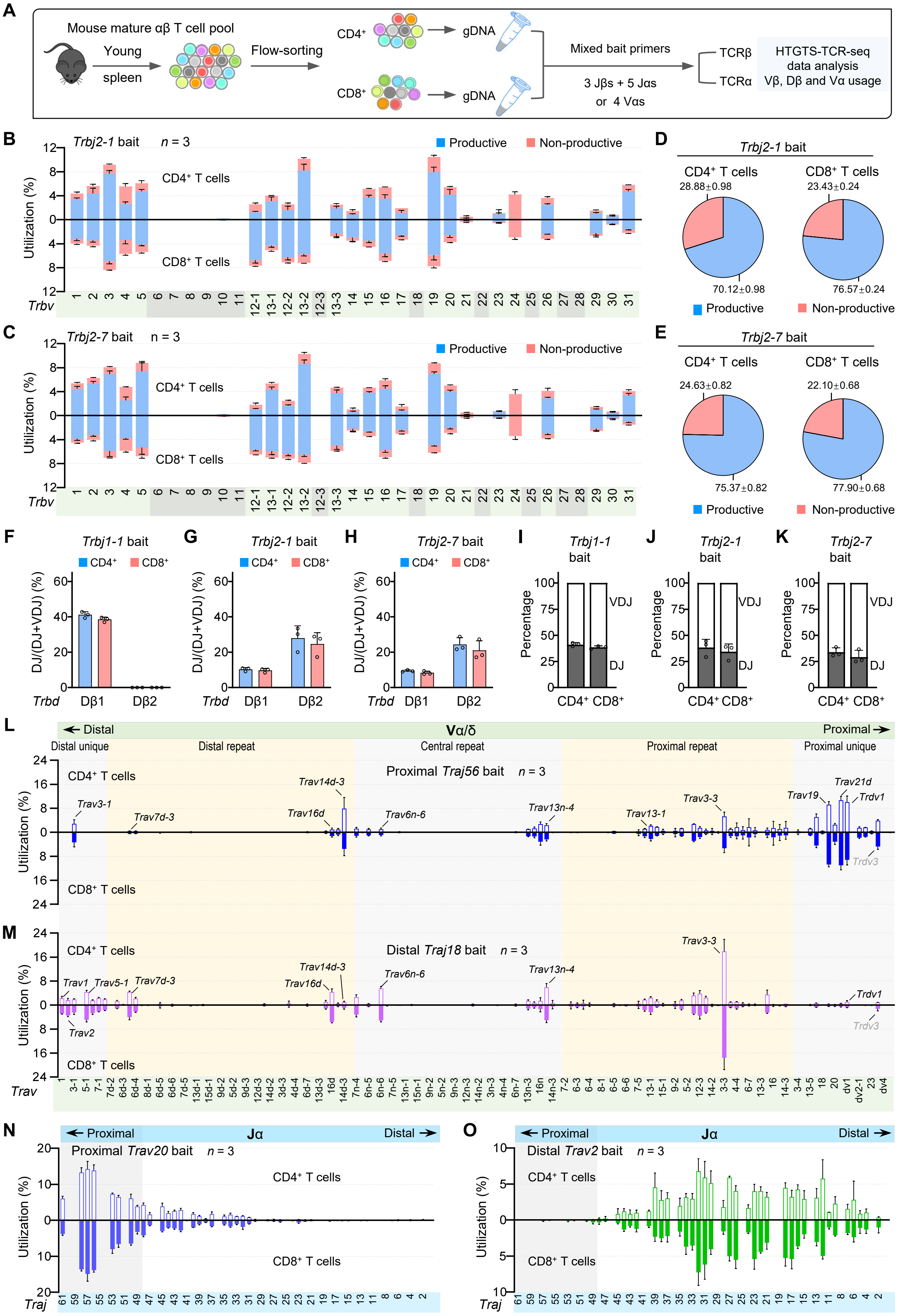

A
Mouse mature αβ T cell pool
Young
spleen
Flow-sorting
CD4+
CD8+
gDNA
Mixed bait primers
3 Jβs + 5 Jαs
or 4 Vαs
TCRβ
TCRα
HTGTS-TCR-seq data analysis
Vβ, Dβ and Vα usage
B
Trbj2-1 bait n = 3
Productive
Non-productive
CD4+ T cells
CD8+ T cells
Utilization (%)
Trbv
C
Trbj2-7 bait n = 3
D
Trbj2-1 bait
CD4+ T cells
CD8+ T cells
28.88±0.98
70.12±0.98
23.43±0.24
76.57±0.24
E
Trbj2-7 bait
24.63±0.82
75.37±0.82
22.10±0.68
77.90±0.68
F
Trbj1-1 bait
G
Trbj2-1 bait
H
Trbj2-7 bait
DJ/(DJ+VDJ) (%)
Trbd
Dβ1
Dβ2
I
Trbj1-1 bait
J
Trbj2-1 bait
K
Trbj2-7 bait
Percentage
VDJ
DJ
L
Distal
Vα/δ
Proximal
Distal unique
Distal repeat
Central repeat
Proximal repeat
Proximal unique
Proximal Traj56 bait n = 3
M
Distal Traj18 bait n = 3
Trav
N
Proximal
Jα
Distal
Proximal Trav20 bait n = 3
Traj
O
Distal Trav2 bait n = 3

Figure S5. Comparison analysis of TCR repertoires in mature CD4$^+$ and CD8$^+$ T cells isolated from the spleens of young C57BL/6 mice (Related to Figure 4). (**A**) Overview of the HTGTS-TCR-seq experimental design for profiling the TCRβ and TCRα repertoires with indicated mixed bait primers in mature CD4$^+$ and CD8$^+$ T cells from young C57BL/6 mice. (**B-C**) Vβ repertoire analysis with productive and nonproductive information from VβDJβ rearrangements in CD4$^+$ (up) and CD8$^+$ (down) T cells using Jβ bait primers *Trbj2-1* (**B**) and *Trbj2-7* (**C**). Pseudogenes are denoted with grey background shading. (**D-E**) Pie charts illustrating the average overall percentage of productive versus nonproductive VβDJβ rearrangements in CD4$^+$ (left) and CD8$^+$ (right) T cells, as derived from the libraries shown in panels (**B-C**). (**F-H**) Comparison analysis of Dβ segment usage within DβJβ rearrangements in CD4$^+$ (blue) and CD8$^+$ (red) T cells, as revealed with bait primers *Trbj1-1* (**F**), *Trbj2-1* (**G**), and *Trbj2-7* (**H**). (**I-K**) Comparison of DJβ: VβDJβ ratios in CD4$^+$ and CD8$^+$ T cells, determined using bait primers *Trbj1-1* (**I**), *Trbj2-1* (**J**), and *Trbj2-7* (**K**). Data in panels (**F-K**) correspond to the libraries analyzed in panels (**B-C**) and Figure 4 panel (**B**). (**L-M**) Vα segment usage in VαJα rearrangements from CD4$^+$ (up) and CD8$^+$ (down) T cells using Jα bait primers *Traj56* (**L**) and *Traj18* (**M**). For comparison, several Vα segments are highlighted. (**N-O**) Jα segment usage in VαJα rearrangements in CD4$^+$ (up) and CD8$^+$ (down) T cells using proximal Vα bait primer *Trav20* (**N**) and *Trav2* (**O**). Data are presented as mean ± s.d.; *n*, number of mice.

Figure. S6

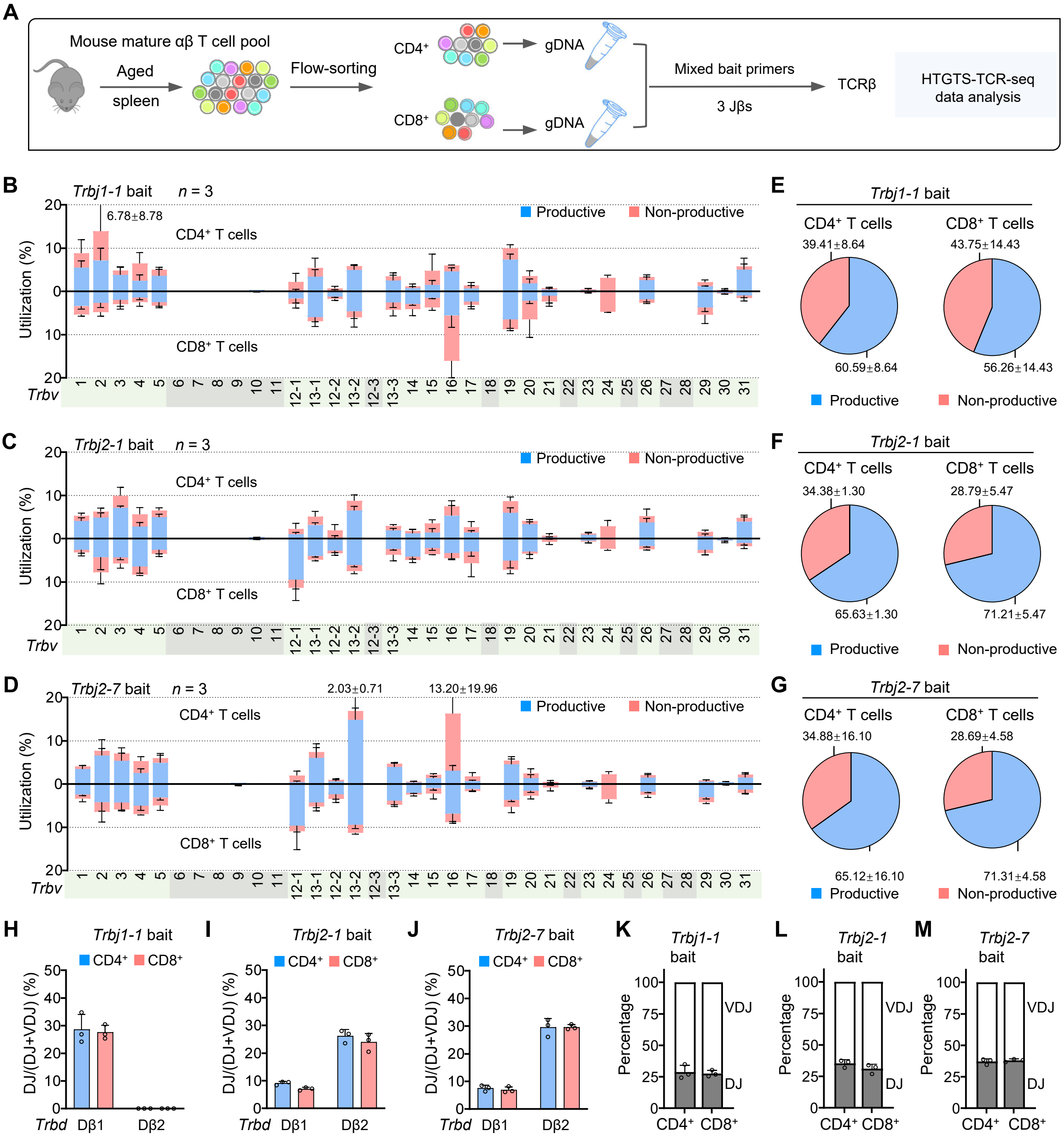

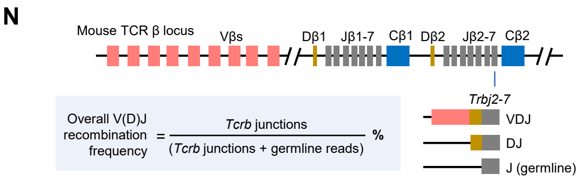

O

Recombination frequency (%)

Bait primer	Cell types	Aged	Young	*P* value
	Thymocytes	23.26±2.71	26.44±2.56	0.2144
Trbj2-7	CD4⁺ T cells	23.72±3.22	35.02±12.45	0.2530
	CD8⁺ T cell	22.06±0.65	30.24±1.93	0.0114

Figure S6. HTGTS-TCR-seq analysis of TCRβ repertoires in mature $CD4^+$ and $CD8^+$ T cells isolated from the spleens of aged C57BL/6 mice. (**A**) Overview of the HTGTS-TCR-seq experimental design for profiling the TCRβ repertoires with indicated mixed bait primers in mature $CD4^+$ and $CD8^+$ T cells from aged C57BL/6 mice. (**B-D**) Vβ repertoire analysis with productive and nonproductive information from VβDJβ rearrangements in $CD4^+$ (up) and $CD8^+$ (down) T cells using Jβ bait primers *Trbj1-1* (**B**), *Trbj2-1* (**C**), and *Trbj2-7* (**D**). Pseudogenes are denoted with grey background shading. (**E-G**) Pie charts illustrating the average overall percentage of productive versus nonproductive VβDJβ rearrangements in $CD4^+$ (left) and $CD8^+$ (right) T cells, as derived from the libraries shown in panels (**B-D**). (**H-J**) Comparison analysis of Dβ segment usage within DβJβ rearrangements in $CD4^+$ (blue) and $CD8^+$ (red) T cells, as revealed with bait primers *Trbj1-1* (**H**), *Trbj2-1* (**I**), and *Trbj2-7* (**J**). (**K-M**) Comparison of DJβ: VβDJβ ratios in $CD4^+$ and $CD8^+$ T cells, determined using bait primers *Trbj1-1* (**K**), *Trbj2-1* (**L**), and *Trbj2-7* (**M**). **(N)** Schematic representation of the strategy used to assess recombination frequency by HTGTS-TCR-seq. The most distal Jβ bait primer, *Trbj2-7*, undergoes only a single recombination event, which remains in the genome regardless of whether the junction is productive or non-productive. This feature allows *Trbj2-7* to serve as an internal control in the HTGTS assay, capturing both V(D)J junctions and germline reads. **(O)** Recombination frequencies assessed using *Trbj2-7* in thymocytes, splenic (SP) $CD4^+$ T cells and $CD8^+$ T cells from young and aged mice. Data are presented as mean ± s.d. ($n = 3$). Unpaired two-sided Student's t-test was used for statistical analysis ($P \leqslant 0.05$, $P \leqslant 0.01$, $P \leqslant 0.001$, $P \leqslant 0.0001$).

Figure. S7

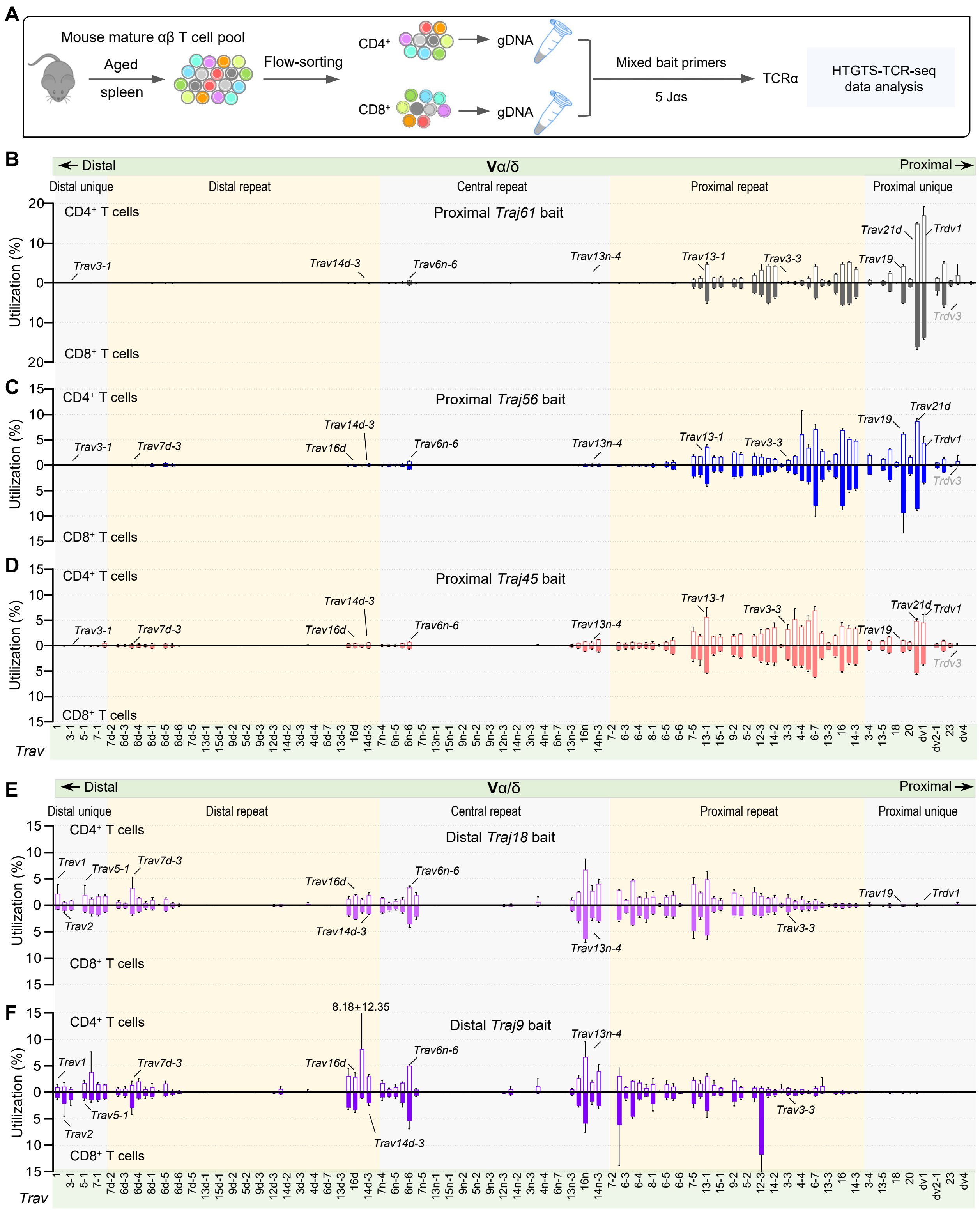
A
Mouse mature αβ T cell pool
Aged
spleen
Flow-sorting
CD4+
CD8+
gDNA
gDNA
Mixed bait primers
5 Jαs
TCRα
HTGTS-TCR-seq data analysis
B
Distal
Vα/δ
Proximal
Distal unique
Distal repeat
Central repeat
Proximal repeat
Proximal unique
CD4+ T cells
CD8+ T cells
Utilization (%)
Proximal Traj61 bait
Trav3-1
Trav14d-3
Trav6n-6
Trav13n-4
Trav13-1
Trav3-3
Trav19
Trav21d
Trdv1
Trdv3
C
Proximal Traj56 bait
Trav7d-3
Trav16d
D
Proximal Traj45 bait
Trav
E
Distal Traj18 bait
Trav1
Trav2
Trav5-1
F
Distal Traj9 bait
8.18±12.35

Figure S7. HTGTS-TCR-seq analysis of *Tcra* gene rearrangement in mature CD4$^+$ and CD8$^+$ T cells isolated from the spleens of aged C57BL/6 mice. (**A**) Overview of the HTGTS-TCR-seq workflow used to profile the TCRα repertoire with indicated mixed bait primers in mature CD4$^+$ and CD8$^+$ T cells from aged C57BL/6 mice. (**B-F**) Vα segment usage in VαJα rearrangements from CD4$^+$ (up) and CD8$^+$ (down) T cells using Jα bait primers *Traj61* (**B**), *Traj56* (**C**), *Traj46* (**D**), *Traj18* (**E**), and *Traj9* (**F**). For comparison, several Vα segments are highlighted. Data are presented as mean ± s.d. (n = 3).

Figure. S8

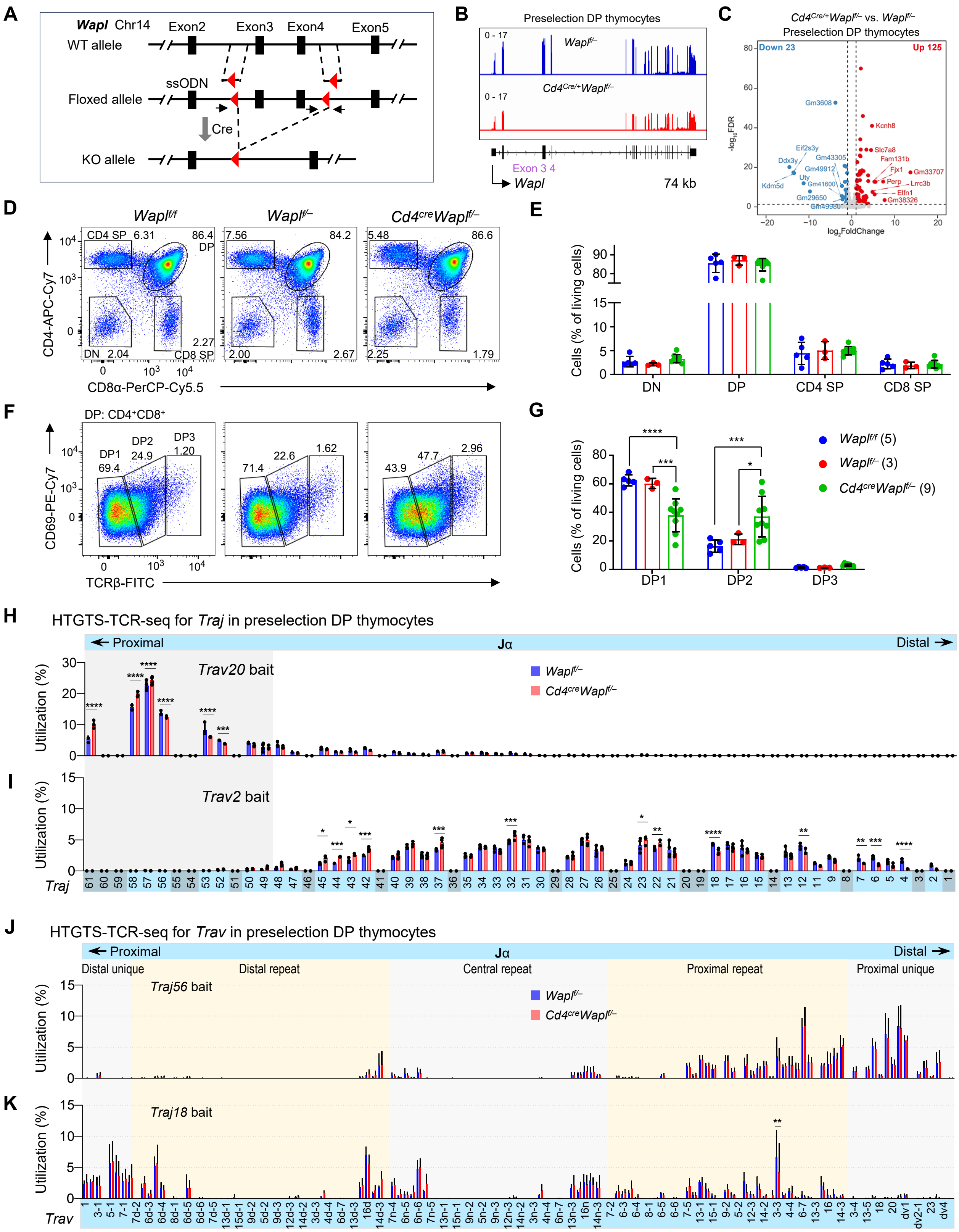

A
Wapl Chr14
WT allele
Exon2
Exon3
Exon4
Exon5
ssODN
Floxed allele
Cre
KO allele
B
Preselection DP thymocytes
Wapl f/–
Cd4 Cre/+ Wapl f/–
Exon 3 4
Wapl
74 kb
C
Cd4 Cre/+ Wapl f/– vs. Wapl f/–
Preselection DP thymocytes
Down 23
Up 125
log2 FoldChange
-log10 FDR
D
Wapl f/f
Wapl f/–
Cd4 cre Wapl f/–
CD4 SP
DP
DN
CD8 SP
CD4-APC-Cy7
CD8α-PerCP-Cy5.5
E
Cells (% of living cells)
DN
DP
CD4 SP
CD8 SP
F
DP: CD4+CD8+
DP1
DP2
DP3
CD69-PE-Cy7
TCRβ-FITC
G
Cells (% of living cells)
DP1
DP2
DP3
Wapl f/f (5)
Wapl f/– (3)
Cd4 cre Wapl f/– (9)
H
HTGTS-TCR-seq for Traj in preselection DP thymocytes
Proximal
Jα
Distal
Trav20 bait
Utilization (%)
I
Trav2 bait
Traj
J
HTGTS-TCR-seq for Trav in preselection DP thymocytes
Distal unique
Distal repeat
Central repeat
Proximal repeat
Proximal unique
Traj56 bait
K
Traj18 bait
Trav

Figure S8. Analysis of thymocyte differentiation and *Tcrα* rearrangements in *Cd4*-cre-mediated WAPL-deficient mice. (**A**) Diagram of the conditional *Wapl* allele with loxP sites (red triangles) flanking exons 3–4. PCR primers locations for genotyping are indicated by horizontal arrows. (**B**) Representative Integrative Genomics Viewer (IGV) tracks displaying results of bulk RNA-seq read distribution of *Wapl* in preselection DP thymocytes from $Cd4^{cre}Wapl^{f/-}$ and control mice, respectively; targeted exons (3–4) highlighted. (**C**) Volcano plots of differentially expressed genes (DEGs) in $Cd4^{cre}Wapl^{f/-}$ and control preselection DP thymocytes. Upregulated (red) or downregulated (blue) are indicated. Two RNA-seq replicates per genotype. (**D-G**) Representative flow cytometry plots (**D**, **F**) of thymocytes from 4–6-week-old $Cd4^{cre}Wapl^{f/-}$ and control mice, and the relative frequencies of the indicated cell types are shown as mean ± s.d. (**E**, **G**). Numbers in the flow cytometry plots indicate the percentage of cells in each gate. Each dot represents an individual mouse. $Wapl^{f/f}$ (n = 5); $Wapl^{f/-}$ (n = 3); $Cd4^{cre}Wapl^{f-/}$ (n = 9); one-way ANOVA followed by Tukey's multiple-comparisons test was performed. (**H-I**) Jα usage quantified by HTGTS-TCR-seq from genomic DNA of preselection DP thymocytes using *Trav20* (**H**) and *Trav2* (**I**) bait primers. (**J-K**) Vα usage quantified by HTGTS-TCR-seq from genomic DNA of preselection DP thymocytes using *Traj56* (**J**) and *Traj18* (**K**) bait primers. Data are represented as the mean ± s.d. (n = 4); two-way ANOVA followed by Tukey's multiple-comparisons test was performed (**H-K**).

Figure. S9

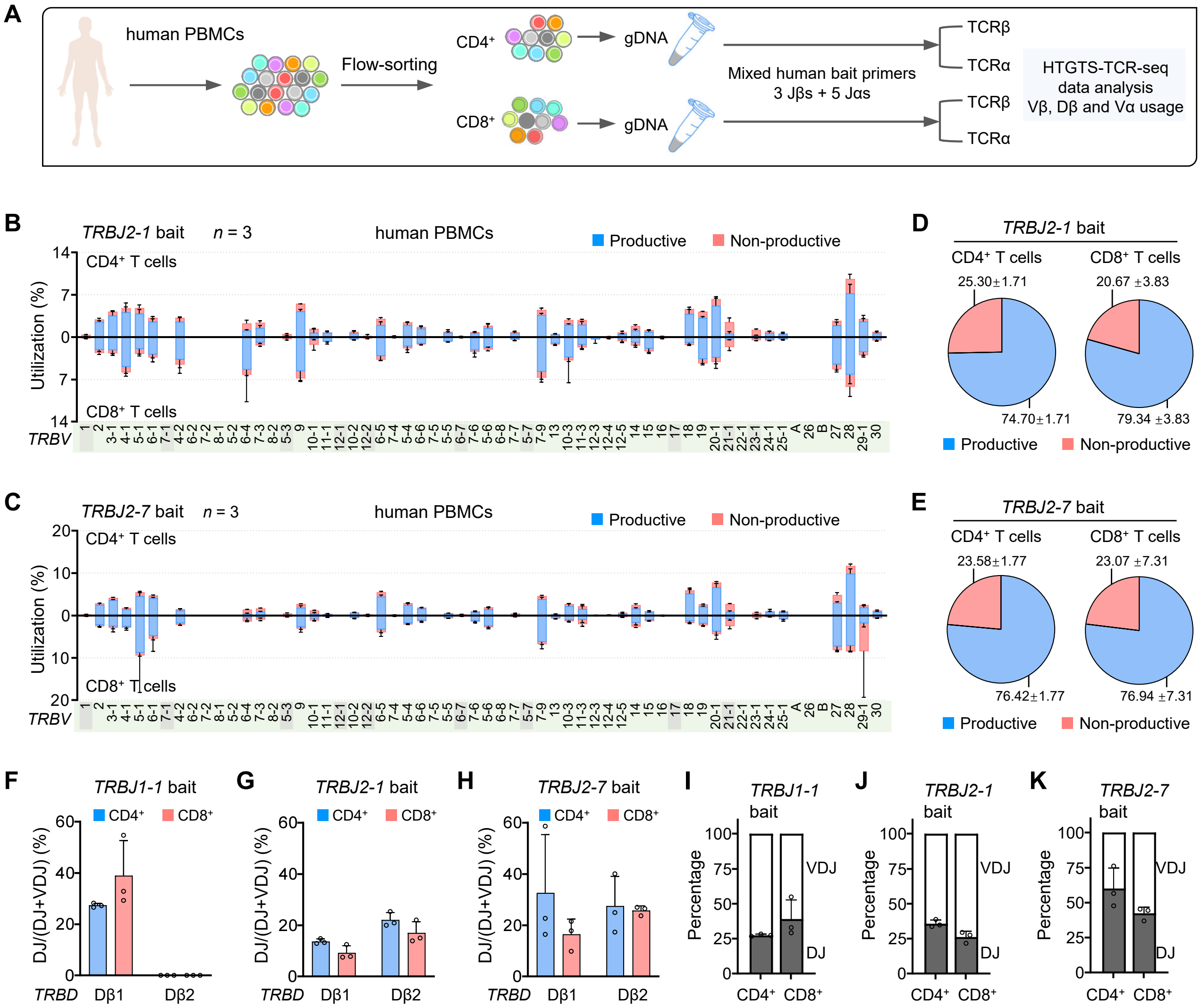
A
human PBMCs
Flow-sorting
CD4+
CD8+
gDNA
gDNA
Mixed human bait primers
3 Jβs + 5 Jαs
TCRβ
TCRα
TCRβ
TCRα
HTGTS-TCR-seq
data analysis
Vβ, Dβ and Vα usage
B
TRBJ2-1 bait n = 3
human PBMCs
Productive
Non-productive
CD4+ T cells
CD8+ T cells
Utilization (%)
TRBV
C
TRBJ2-7 bait n = 3
human PBMCs
Productive
Non-productive
CD4+ T cells
CD8+ T cells
Utilization (%)
TRBV
D
TRBJ2-1 bait
CD4+ T cells
CD8+ T cells
25.30±1.71
20.67 ±3.83
74.70±1.71
79.34 ±3.83
Productive
Non-productive
E
TRBJ2-7 bait
CD4+ T cells
CD8+ T cells
23.58±1.77
23.07 ±7.31
76.42±1.77
76.94 ±7.31
Productive
Non-productive
F
TRBJ1-1 bait
CD4+
CD8+
DJ/(DJ+VDJ) (%)
TRBD Dβ1 Dβ2
G
TRBJ2-1 bait
CD4+
CD8+
DJ/(DJ+VDJ) (%)
TRBD Dβ1 Dβ2
H
TRBJ2-7 bait
CD4+
CD8+
DJ/(DJ+VDJ) (%)
TRBD Dβ1 Dβ2
I
TRBJ1-1 bait
Percentage
VDJ
DJ
CD4+ CD8+
J
TRBJ2-1 bait
Percentage
VDJ
DJ
CD4+ CD8+
K
TRBJ2-7 bait
Percentage
VDJ
DJ
CD4+ CD8+

Figure S9. Comparison analysis of TCR repertoires in human mature αβ T cells (Related to Figure 5). (**A**) Overview of the HTGTS-TCR-seq experimental design for profiling the TCRβ and TCRα repertoires in human $CD4^+$ and $CD8^+$ T cells. (**B-C**) Vβ repertoire profiles with productive and nonproductive information from VβDJβ rearrangements in $CD4^+$ (up) and $CD8^+$ (down) T cells using Jβ bait primers *TRBJ2-1* (**B**) and *TRBJ2-7* (**C**). Pseudogenes are denoted with grey background shading. (**D-E**) Pie charts showing the average overall percentage of productive versus nonproductive VβDJβ rearrangements derived from libraries shown in panels **(B-C)** for $CD4^+$ (left) and $CD8^+$ (right) T cells. (**F-H**) Comparison analysis of Dβ segment usage within DJβ rearrangements in $CD4^+$ (blue) and $CD8^+$ (red) T cells, as revealed with bait primers *TRBJ1-1* (**F**), *TRBJ2-1* (**G**), and *TRBJ2-7* (**H**). (**I-K**) Comparison of DJβ:VβDJβ ratios in $CD4^+$ and $CD8^+$ T cells, determined using bait primers *TRBJ1-1* (**I**), *TRBJ2-1* (**J**), and *TRBJ2-7* (**K**). Data in panels (**F-K**) correspond to the libraries analyzed in panel (**B-C**) and Figure 5 panels (**B**). Data are presented as mean ± s.d.; *n*, number of donors.

Figure. S10

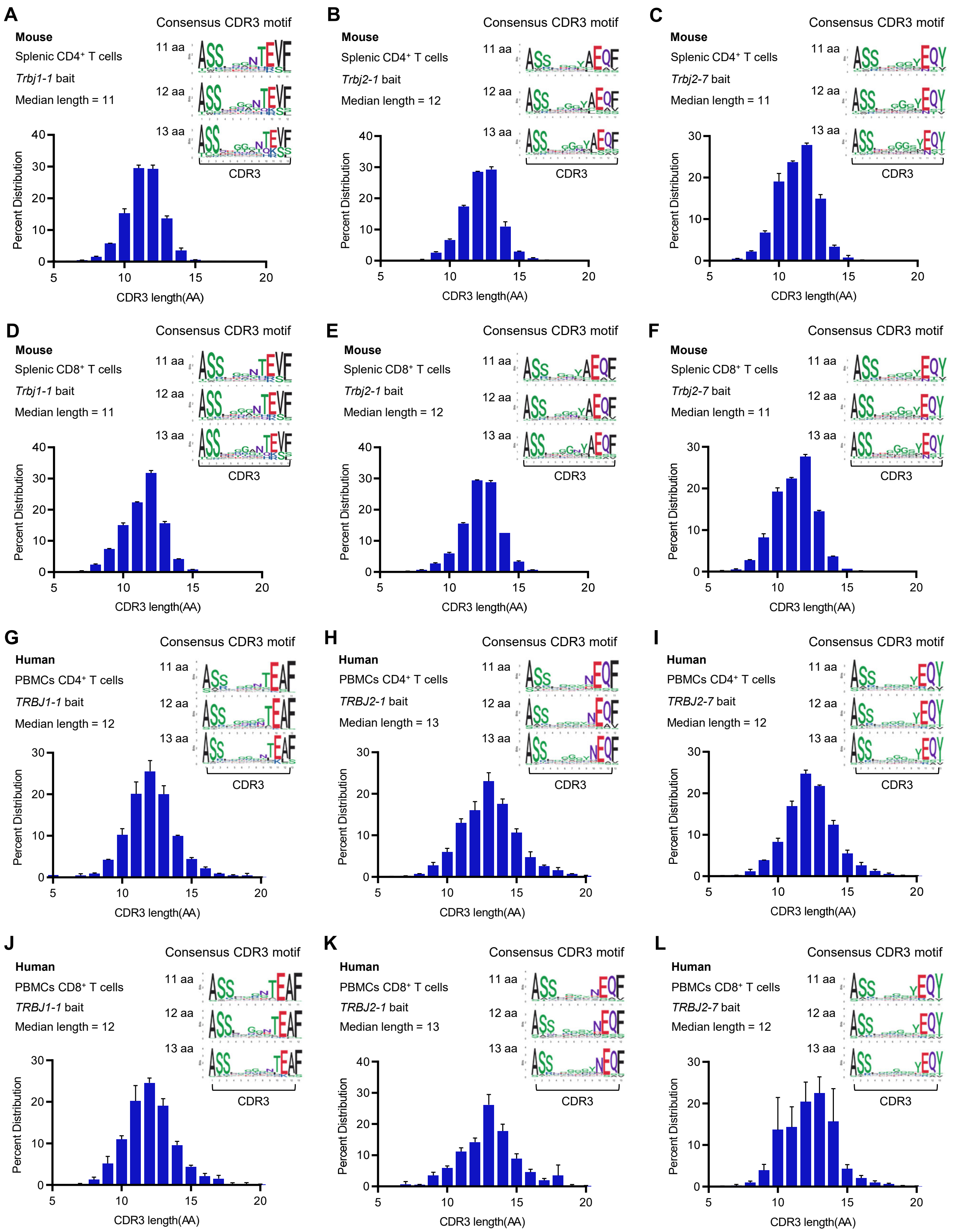
A
Mouse
Splenic CD4⁺ T cells
Trbj1-1 bait
Median length = 11
Consensus CDR3 motif
11 aa
12 aa
13 aa
CDR3
Percent Distribution
CDR3 length(AA)
B
Mouse
Splenic CD4⁺ T cells
Trbj2-1 bait
Median length = 12
Consensus CDR3 motif
CDR3
C
Mouse
Splenic CD4⁺ T cells
Trbj2-7 bait
Median length = 11
Consensus CDR3 motif
CDR3
D
Mouse
Splenic CD8⁺ T cells
Trbj1-1 bait
Median length = 11
Consensus CDR3 motif
E
Mouse
Splenic CD8⁺ T cells
Trbj2-1 bait
Median length = 12
Consensus CDR3 motif
F
Mouse
Splenic CD8⁺ T cells
Trbj2-7 bait
Median length = 11
Consensus CDR3 motif
G
Human
PBMCs CD4⁺ T cells
TRBJ1-1 bait
Median length = 12
Consensus CDR3 motif
H
Human
PBMCs CD4⁺ T cells
TRBJ2-1 bait
Median length = 13
Consensus CDR3 motif
I
Human
PBMCs CD4⁺ T cells
TRBJ2-7 bait
Median length = 12
Consensus CDR3 motif
J
Human
PBMCs CD8⁺ T cells
TRBJ1-1 bait
Median length = 12
Consensus CDR3 motif
K
Human
PBMCs CD8⁺ T cells
TRBJ2-1 bait
Median length = 13
Consensus CDR3 motif
L
Human
PBMCs CD8⁺ T cells
TRBJ2-7 bait
Median length = 12
Consensus CDR3 motif

Figure S10. HTGTS-TCR-seq analysis of CDR3 features in mouse and human peripheral $CD4^+$ T cells. (**A-C**) Length distribution of productive VβDβJβ CDR3 regions with consensus motif analysis of 11-13 amino acids in mouse splenic $CD4^+$ T cell repertoire using bait primers *Trbj1-1* (**A**), *Trbj2-1* (**B**), and *Trbj2-7* (**C**). (**D-F**) Length distribution of productive VβDβJβ CDR3 regions with consensus motif analysis of 11-13 amino acids in mouse splenic $CD8^+$ T cell repertoire using bait primers *Trbj1-1* (**D**), *Trbj2-1* (**E**), and *Trbj2-7* (**F**). Data are presented as mean ± s.d.; *n*, number of mice (**A-F**). (**G-I**) Length distribution of productive VβDβJβ CDR3 regions with consensus motif analysis of 11-13 amino acids in human peripheral $CD4^+$ T cell repertoire using bait primers *TRBJ1-1* (**G**), *TRBJ2-1* (**H**), and *TRBJ2-7* (**I**). (**J-L**) Length distribution of productive VβDβJβ CDR3 regions with consensus motif analysis of 11-13 amino acids in human peripheral $CD8^+$ T cell repertoire using bait primers *TRBJ1-1* (**J**), *TRBJ2-1* (**K**), and *TRBJ2-7* (**L**). Data are presented as mean ± s.d.; *n*, number of donors (**G-L**).

Figure. S11

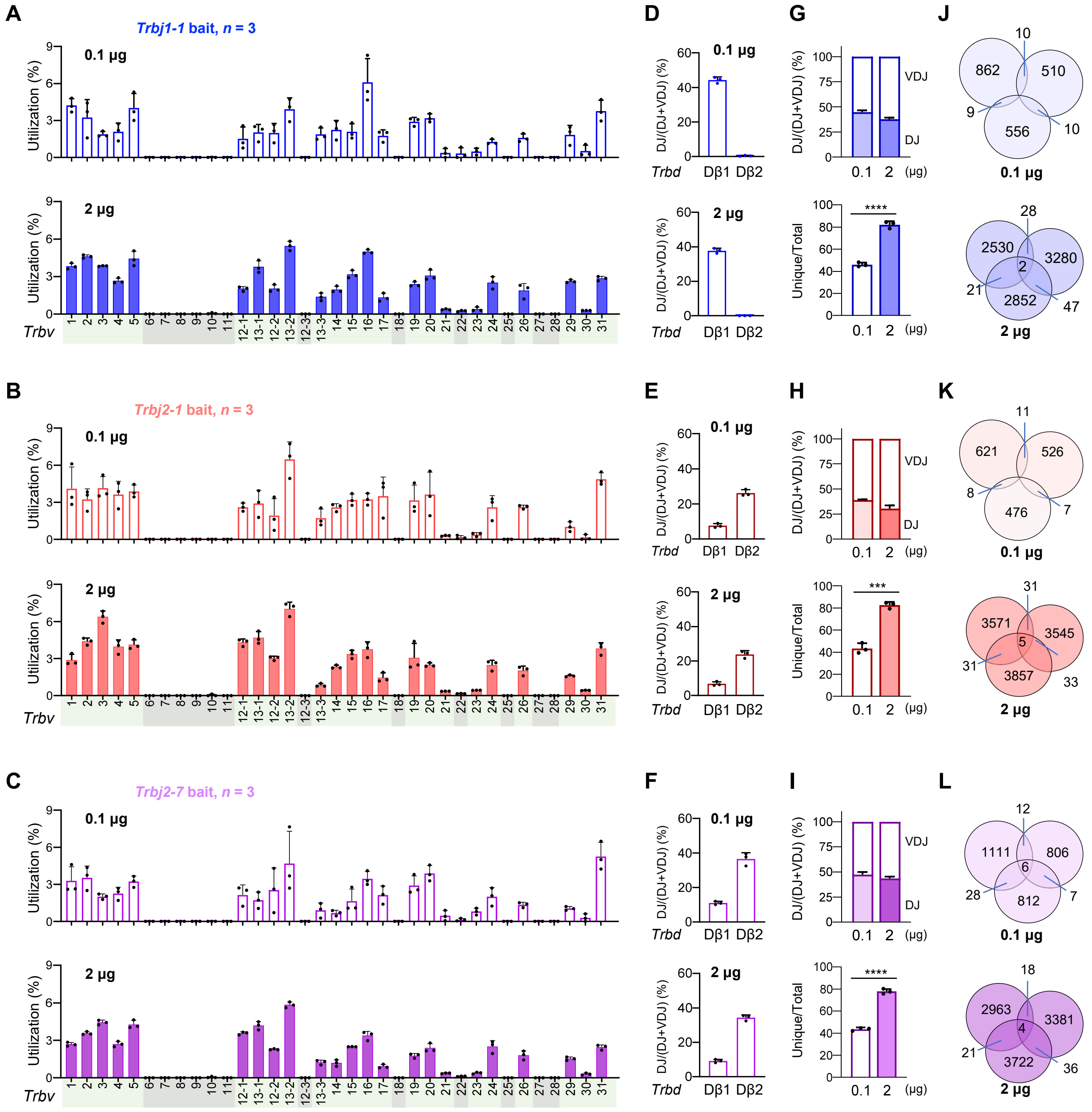
A
Trbj1-1 bait, n = 3
0.1 µg
2 µg
Utilization (%)
Trbv
D
0.1 µg
2 µg
DJ/(DJ+VDJ) (%)
Trbd
Dβ1 Dβ2
G
DJ/(DJ+VDJ) (%)
VDJ
DJ
Unique/Total
****
0.1 2 (µg)
J
10
862
510
9
556
10
0.1 µg
28
2530
3280
2
21
2852
47
2 µg
B
Trbj2-1 bait, n = 3
E
H
***
K
11
621
526
8
476
7
31
3571
3545
5
31
3857
33
C
Trbj2-7 bait, n = 3
F
I
****
L
12
1111
806
6
28
812
7
18
2963
3381
4
21
3722
36

Figure S11. Characterization of mouse TCRβ repertoire generated from different amounts of starting genomic DNA. (**A-C**) Comparison analysis of the Vβ segment usage within *Tcrb* gene rearrangements using three different bait primers: *Trbj1-1* (**A**), *Trbj2-1* (**B**), and *Trbj2-7* (**C**). Libraries were generated using splenic $CD3^+$ T cells, with different amounts of genomic DNA (0.1 μg and 2 μg). Pseudogenes are denoted with grey background shading. (**D-F**) Analysis of Dβ segment usage within DβJβ rearrangements using different starting materials, as revealed with bait primers *Trbj1-1* (**D**), *Trbj2-1* (**E**), and *Trbj2-7* (**F**). (**G-I**) Comparison of DβJβ: VβDβJβ ratios (up) and proportion of unique CDR3 sequences (down) for libraries generated from different amounts of starting materials, determined using bait primer *Trbj1-1* (**G**), *Trbj2-1* (**H**), and *Trbj2-7* (**I**). (**J-L**) Venn plot showing the number of identical CDR3 sequences between libraries at varying amounts of starting material using bait primer *Trbj1-1* (**J**), *Trbj2-1* (**K**), and *Trbj2-7* (**L**).
